# Human apoA-I[Lys107del] mutation affects lipid surface behavior of apoA-I and its ability to form large nascent HDL

**DOI:** 10.1016/j.jlr.2022.100319

**Published:** 2022-12-13

**Authors:** Irina N. Gorshkova, Nathan L. Meyers, Haya Herscovitz, Xiaohu Mei, David Atkinson

**Affiliations:** Department of Physiology and Biophysics, Boston University School of Medicine, Boston, MA, USA

**Keywords:** Apolipoprotein A-I, lipoproteins, ABCA1, cholesterol/efflux, natural/mutation, HDL-C, α-helix, drop tensiometry, DMPC, 1,2-dimyristoylphosphatidylcholine, GM1, ganglioside, monosialotetrahexosylganglioside, nHDL, nascent HDL, RCT, reverse cholesterol transport, SEC, size exclusion chromatography, SUV, small unilamellar vesicles, TFE, trifluoroethanol, TG, triglyceride, TO, triolein, TO/W, triolein / water

## Abstract

Population studies have found that a natural human apoA-I variant, apoA-I[K107del], is strongly associated with low HDL-C but normal plasma apoA-I levels. We aimed to reveal properties of this variant that contribute to its unusual phenotype associated with atherosclerosis. Our oil-drop tensiometry studies revealed that compared to WT, recombinant apoA-I[K107del] adsorbed to surfaces of POPC-coated triolein drops at faster rates, remodeled the surfaces to a greater extent, and was ejected from the surfaces at higher surface pressures on compression of the lipid drops. These properties may drive increased binding of apoA-I[K107del] to and its better retention on large triglyceride-rich lipoproteins, thereby increasing the variant’s content on these lipoproteins. While K107del did not affect apoA-I capacity to promote ABCA1-mediated cholesterol efflux from J774 cells, it impaired the biogenesis of large nascent HDL particles resulting in the formation of predominantly smaller nascent HDL. Size-exclusion chromatography of spontaneously reconstituted 1,2-dimyristoylphosphatidylcholine-apoA-I complexes showed that apoA-I[K107del] had a hampered ability to form larger complexes but formed efficiently smaller-sized complexes. CD analysis revealed a reduced ability of apoA-I[K107del] to increase α-helical structure on binding to 1,2-dimyristoylphosphatidylcholine or in the presence of trifluoroethanol. This property may hinder the formation of large apoA-I[K107del]-containing discoidal and spherical HDL but not smaller HDL. Both factors, the increased content of apoA-I[K107del] on triglyceride-rich lipoproteins and the impaired ability of the variant to stabilize large HDL particles resulting in reduced lipid:protein ratios in HDL, may contribute to normal plasma apoA-I levels along with low HDL-C and increased risk for CVD.

ApoA-I is a major protein component of HDL and is also present in large triglyceride (TG)-rich lipoproteins, VLDL, and chylomicrons (1, 2). Plasma levels of HDL-C and apoA-I are inversely related to the risk of CVD and atherosclerosis (3). However, pharmacological treatments that increased HDL-C did not result in significant reduction of clinical cardiovascular events [(4, 5) and references cited therein]. This outcome supports the concept that correct properties of HDL are vital for their cardioprotective functions (4, 5, 6). The cardioprotective functions of HDL mainly relate to its involvement in the pathways of reverse cholesterol transport (RCT), with apoA-I being involved in the critical steps of these pathways (5, 6, 7). Interaction of apoA-I with ABCA1 promotes cellular cholesterol efflux and results in the formation of discoidal nascent HDL (nHDL). Maturation of nHDL into spherical particles involves LCAT-mediated cholesterol esterification, with apoA-I being a principal activator of the enzyme. Interaction of HDL-bound apoA-I with hepatic scavenger receptor B1 promotes extraction of cholesteryl ester from the circulation. Mutations in apoA-I affect the protein stability and metabolism and thereby, plasma concentrations of apoA-I and HDL (8). Many of the naturally occurring apoA-I mutations are associated with low levels of both plasma apoA-I and HDL-C and with increased risk for CVD and atherosclerosis (http://www.hgmd.cf.ac.uk/ac/gene.php?gene=APOA1). Population studies also demonstrated a strong association of low HDL-C levels with low plasma apoA-I levels (9).

However, two large population studies, Copenhagen City Heart and Copenhagen General Population Studies (10), found that K107del mutation of apoA-I (apoA-I[K107del]), a naturally occurring apoA-I heterozygous variant with a deletion of Lysine 107, was strongly associated with low plasma concentrations of HDL-C but normal levels of apoA-I. Reduced plasma HDL-C without a corresponding reduction in plasma apoA-I concentrations were also reported earlier in subjects with apoA-I[K107del] in Finland and Germany (11, 12). Such dissociation between plasma concentrations of apoA-I and HDL-C seems especially surprising for the apoA-I[K107del] variant that is also associated with hereditary amyloidosis (13, 14), a disease characterized by enhanced proteolysis of mutated apoA-I and deposition of protein fragments as fibrils in vital organs that lead to removal of apoA-I from the circulation. Previous studies have shown that K107del leads to apoA-I destabilization (15, 16), enhanced fractional catabolism (11), and reduced binding to HDL (17). Each of these properties of apoA-I[K107del] should also lead to reduced plasma apoA-I concentrations. Nevertheless, the K107del mutation was not found to be associated with reduced plasma apoA-I concentrations (10, 11), implying that some compensatory mechanisms may exist that maintain normal plasma apoA-I levels in subjects carrying the mutation. In this study, we investigated potential molecular mechanisms that may contribute to the reduced HDL-C coupled with normal apoA-I concentrations in plasma of subjects with the K107del mutation. Insights into the causes of this unusual phenotype are important for better understanding the function of the apoA-I[K107del] variant that is also linked to atherosclerosis (13), premature coronary heart disease (18), and elevated plasma TG (12).

One of the mechanisms contributing to the normal plasma apoA-I levels in carriers of the apoA-I[K107del] variant may relate to the increased apoA-I production into HDL containing apoA-I without apoA-II (but not in HDL containing both apoA-I and apoA-II) (11). However, apoA-I has increased catabolic rate of both HDL subspecies in individuals carrying the K107del mutation (11, 19). Another factor contributing to the reduced HDL-C coupled with normal plasma apoA-I levels may be an increased fraction of apoA-I on TG-rich lipoproteins along with reduced plasma HDL concentrations. ApoA-I can exist in plasma in lipid-free, lipid-poor, and lipid-bound states and exchange among lipoproteins (20). Our earlier in vitro studies showed that incubation of WT or apoA-I[K107del] with TG-POPC synthetic emulsion particles that mimic VLDL-sized particles resulted in more molecules of the mutant bound to the particles compared to WT (15). Contrarily, when WT or pro-apoA-I[K107del] were incubated with the lipoprotein fraction of plasma, a smaller amount of the mutant, compared to WT, bound to HDL (17). These observations suggest that compared to WT, apoA-I[K107del] may have a higher affinity to TG-rich lipoproteins, but a lower affinity to HDL, despite the fact that in plasma, the majority of apoA-I is bound to HDL. It is not clear why the K107 deletion may affect apoA-I binding to larger VLDL and to smaller HDL in opposite ways. To look into the factors that may affect binding of apoA-I[K107del] to lipoprotein surfaces, we studied the surface behavior of apoA-I[K107del] at triolein (TO)/water (W) and POPC/TO/W interfaces using oil-drop tensiometry (21, 22, 23, 24, 25, 26). TO is a common TG, and POPC is the most abundant phospholipid in lipoproteins. The TO/W interface provides a model for protein interactions with a hydrophobic TG core, a major component of TG-rich lipoproteins, while POPC/TO/W interfaces model the surface of TG-rich lipoproteins (24, 25). Apolipoprotein adsorption to and desorption from the lipid surfaces in response to surface pressure changes is thought to mirror apolipoprotein binding to and dissociation from lipoproteins in vivo (21, 22, 23, 24, 25). These surface pressure changes occur throughout the lipoprotein remodeling involving LCAT and lipase reactions, as well as transfer of TG, cholesteryl ester, and phospholipid molecules between lipoproteins mediated by plasma lipid transfers proteins (20, 24, 25).

We also investigated the effects of the K107del mutation on the biogenesis of nHDL promoted by apoA-I during ABCA1-mediated cholesterol efflux from J774 cells. It has been shown earlier that deletion of K107 in apoA-I does not affect net ABCA1-mediated cholesterol efflux from various cell lines (17, 27, 28). In this study, we looked for the first time into the effect of the K107del mutation on the size distribution of nHDL, as changes in nHDL sizes may contribute to the altered plasma ratio of apoA-I to HDL-C. As we found that deletion of K107 led to impaired formation of large and very large nHDL particles, we wanted to probe a structural basis of the altered size distribution of nHDL. To that end, we investigated the effects of the K107 deletion on the formation of dimyristoylphosphatidylcholine (DMPC)-apoA-I complexes at various lipid:protein ratios and the ability of apoA-I to form additional α-helical structure in the presence of a helical structure inducer, trifluoroethanol (TFE).

## Materials and Methods

### Reagents

POPC (25 mg/ml in chloroform), DMPC, TO, and BODIPY-cholesterol (TopFluor) were purchased from Avanti Polar Lipids (Alabaster, AL). TFE (extra pure) was purchased from Thermo Fisher Scientific. CodonPlus-RIL cells and the QuickChange mutagenesis kit were purchased from Stratagene (LaJolla, CA). Media (RPMI and MEM), fetal bovine serum, and penicillin streptomycin were from Invitrogen (Waltham, MA), CPT-cAMP (cAMP), HRP-conjugated-cholera toxin subunit B, and protease inhibitors were from Sigma. Polyclonal goat antibodies to apoA-I were purchased from Calbiochem and Invitrogen. Polyclonal antibodies against ABCA1 were from Novus Biologicals (Littleton, CO), and monoclonal antibodies against pan actin were from Cytoskeleton Inc. (Denver, CO).

### ApoA-I expression and purification

WT apoA-I (WT) and apoA-I[K107del] were expressed, purified, and stored as described previously (15). Before each experiment, a protein aliquot was thawed and freshly refolded by dialysis against 4M guanidine hydrochloride followed by extensive dialysis against an appropriate buffer: 2 mM sodium phosphate, pH 7.4, for oil-drop tensiometry experiments; TBS, pH 7.4, for formation of DMPC-apoA-I complexes, or 10 mM sodium phosphate, pH 7.6, for all other experiments.

### Drop-tensiometry

#### Interfacial tension measurements

An I.T. Concept Tracker oil-drop tensiometer (Longessaigne, France) was used to measure interfacial tension (γ) (21, 22, 23, 25). Experiments were carried out at 25 ± 0.1°C in a thermostated system and repeated at least twice. To measure γ of the TO/W interface, TO drops of 16 μl were formed in a cuvette containing 6 ml of gently stirred standard buffer with a given amount of protein, and γ was monitored continuously until it reached equilibrium.

For the experiments with POPC/TO/W interfaces, small unilamellar vesicles (SUVs) were prepared from POPC solution in chloroform by drying the chloroform under nitrogen and resuspending the dried lipid film in standard buffer (2 mM sodium phosphate, pH 7.4) to a concentration of 2.5–5 mg/ml. The lipid suspension was then sonicated for 60 min with pulsed duty cycle of 30%. The resulting suspension was virtually clear and contained SUV particles with a diameter of 21–28 nm as estimated by negative staining electron microscopy. To measure γ of the POPC/TO/W interface, a 16 μl TO drop was formed in 6 ml gently stirred standard buffer containing 0–1 mg of POPC SUV stock and γ was recorded continuously. Adsorption of POPC molecules onto the TO drop lowered γ. After POPC was adsorbed, the excess POPC vesicles were removed from the aqueous phase by the buffer exchange procedure as described (26), and γ stayed constant. After TO/W interfaces had stabilized at an initial γ (γ_i_) of about 32 mN/m or POPC/TO/W interfaces reached γ_i_ of about 25 mN/m, varied amounts of WT or apoA-I[K107del] were added to the aqueous phase to achieve different protein concentrations in the range 1.3–5.3 × 10^-7^M. The γ of the interface was recorded continuously until it reached an equilibrium value (γ_eq_).

#### Penetration of the proteins into the POPC/TO/W interface and measurement of exclusion pressure

To compare the ability of WT and apoA-I[K107del] to penetrate the POPC/TO/W interface, the adsorption isotherms were measured as described above after the initial surface pressure of the interface (Π_i_) was set at various target values. In short, after the TO drop (16 μl) was coated with POPC and γ reached ∼25 mN/m, the excess POPC vesicles were removed from the aqueous phase. Then the interface was either expanded or compressed as described (23), resulting in a decrease or increase of the surface concentration of POPC (ᴦ_POPC_) and the corresponding changes in Π_i_. Values for Π_i_ were obtained from γ of a TO/W interface (γ_TO_ = 32 mN/m) minus γ of the interface with POPC (Π_i_ = γ_TO_ – γ_i_). Larger values of ᴦ_POPC_ lead to smaller γ_i_ and larger Π_i_. An aliquot of protein was then added to the aqueous phase (at final concentration of about 6 × 10^−8^M), γ_eq_ was measured, and equilibrium pressure Π_eq_ was obtained (Π_eq_ = γ_TO_ – γ_eq_). The values of the increase in surface pressure (ΔΠ = Π_eq_ - Π_i_) were plotted against Π_i_ and the data were fit to a linear regression. A linear extrapolation of the plot gave values of exclusion pressure (Π_EX_) at the x-intercept where ΔΠ = 0. At a surface pressure equal to or higher than Π_EX_, protein cannot penetrate the POPC/TO/W interface.

#### Slow expansion and compression of the POPC/TO/W interface and measurement of envelope (retention) pressure

Slow expansion and compression of the POPC/TO/W interfaces were performed to estimate values of retention pressure at which protein begins to be ejected from the surface upon compression (26). After POPC adsorption to the TO drop lowered γ to about 25 mN/m and buffer was exchanged with POPC-free buffer, Π_i_ was set at various values by increasing or decreasing the drop volume. The values of Π_i_ were used to calculate ᴦ_POPC_ as a percentage of TO drop surface covered by POPC (26). Protein was added to the buffer and adsorption lowered γ to γ_eq_. The protein in the aqueous phase was removed by buffer exchange and the interface was slowly expanded to a volume of 25–28 μl. After γ equilibrated for 200 s or longer, the volume was decreased at the rate 0.02 μl/s with varying lower limit. Pressure-area (Π-A) curves were derived from the compression procedure as previously described (26), with pressure Π calculated from γ when the surface area (A) changed. Each Π-A curve exhibits an envelope point, which marks a change in slope. The envelope point was determined by plotting the values of the derivative dΠ/dA against area A. The envelope point is the surface area (A_ENV_) and pressure (Π_ENV_) at which protein begins to be ejected from a POPC/TO/W interface. The values of Π_ENV_ reflect the retention pressure.

### Cholesterol efflux and nHDL biogenesis

Mouse macrophage-derived J774 cells were maintained in media (RPMI) supplemented with 10% fetal bovine serum, penicillin, and streptomycin. For the experiments, cells were plated in 24-well plates and incubated with BODIPY-cholesterol complexed to methyl ß-cyclodextrin to label the cholesterol pool as described (29, 30). ABCA1 was then upregulated by incubating the cells for 20–24 h in medium A (RPMI supplemented with 0.2% BSA and 2 μg/ml ACAT inhibitor) with or without 0.3 mM CPT-cAMP. Cells were then washed and incubated with either WT or apoA-I[K107del] (10 μg/ml) in medium B (MEM-HEPES containing 0.01% BSA supplemented with 2 μg / ml ACAT inhibitor) containing 0.3 mM cAMP for 6 or 24 h. Control cells were incubated in media lacking acceptors (apoA-I) or cAMP. After the incubation, media were harvested and filtered through 0.22 μm membrane to remove cell debris. Cells were lysed by incubation in ice-cold 1% sodium cholate containing protease inhibitors for 2 h. Fluorescence intensity in media and cell lysates was measured using a plate reader (Tecan Infinite M1000) as described (31). Fluorescence readings were adjusted by subtraction of a background fluorescence in media collected from cells incubated in media lacking acceptors. Efflux was calculated as a percentage of total fluorescence in media and cells. All experiments were carried out in duplicates or triplicates and were repeated 3–5 times.

nHDL particles were detected by resolving the efflux media on 4%–15% native PAGE followed by immunoblotting with antibodies to apoA-I. Bands corresponding to apoA-I-containing nHDL and lipid-free apoA-I were visualized using the ECL system and quantified using KwikQuant image analyzer. The membranes were then stripped with 0.25 M glycine, pH 2.5, for 1 h. Blots were then subjected to ligand blotting using HRP-conjugated cholera toxin subunit B for 30 min to detect the ganglioside, monosialotetrahexosylganglioside (GM1) (32). The membranes were imaged and quantified as described above. ABCA1 in cells was evaluated by resolving cell lysates on 4%–15% SDS-PAGE followed by immunoblotting using antibodies to ABCA1. Actin, the loading control, was detected by probing the membranes using antibodies against pan actin. Bands were visualized and quantified as described above.

### Reconstitution and CD analysis of DMPC-apoA-I complexes

DMPC-apoA-I complexes were obtained by spontaneous reconstitution as previously described (33), with some modifications. To prepare DMPC stock suspension (5 mg/ml), 5 mg DMPC in chloroform was dried under nitrogen in a borosilicate test tube and placed under vacuum overnight. One milliliter of TBS was added to the film of dry lipid and vortexed vigorously for 5 min at room temperature. During this process, the lipid dispersion was heated periodically, 2–3 times, for 30 s to the temperature above the phase transition of DMPC (24°C) to facilitate the multilamellar vesicle preparation. DMPC suspension was mixed with WT or apoA-I[K107del] solutions in TBS in the presence of 0.04% sodium azide to give lipid/protein ratios 2, 4, or 8 (w:w) and apoA-I concentration of 0.1 mg/ml. The mixtures were then incubated at 24°C with gentle shaking for 66 h. WT and apoA-I[K107del] in TBS without DMPC were incubated along. Following the incubation, the samples were eluted through a Superose 6 HP 10/30 column (Pharmacia) using Ӓkta FPLC system, equilibrated with TBS, pH 7.4, at a flow of 0.5 ml/min. The size-exclusion chromatography (SEC) was used to separate DMPC-apoA-I complexes from the remaining lipid-free apoA-I and compare the elution profiles that reflect sizes of the resultant complexes. For each DMPC:apoA-I ratio, the experiment was repeated at least twice using a fresh preparation of DMPC multilamellar vesicles in each case. Fractions containing DMPC-apoA-I complexes were pooled and analyzed by immunoblotting with antibodies to apoA-I following separation of the particles on 4%–15% native PAGE, as described above.

The pooled fractions containing DMPC-apoA-I particles were dialyzed against 10 mM phosphate buffer, concentrated if necessary, and far-UV CD spectra of each sample were recorded at 25°C on Jasco J-1000 CD spectrometer (Jasco Inc., Easton, MD). The α-helical content was determined from the mean residue ellipticity at 222 nm as described (34). Protein concentration in the samples was determined by BCA protein assay (Thermo Scientific) in the presence of 1 mg/ml SDS in the working reagent.

### ApoA-I α-helical content in the presence of TFE

The helical structure inducer, TFE, was used to assess the ability of WT and apoA-I[K107del] to form additional α-helical structure. Various amounts of TFE were added to the protein solutions in 10 mM phosphate buffer to have TFE concentrations 20, 40, 60, or 80% (v/v) and protein concentration 0.04 mg/ml. After incubation of the samples for 1 h at 4°C, CD spectra were recorded at 25°C and the α-helical content was determined as described above. The experiment was repeated three times.

## Results

### Effect of K107 deletion on apoA-I affinity for lipid/water interfaces

#### Protein adsorption to TO/W and POPC/TO/W interfaces

**Fig. 1:**
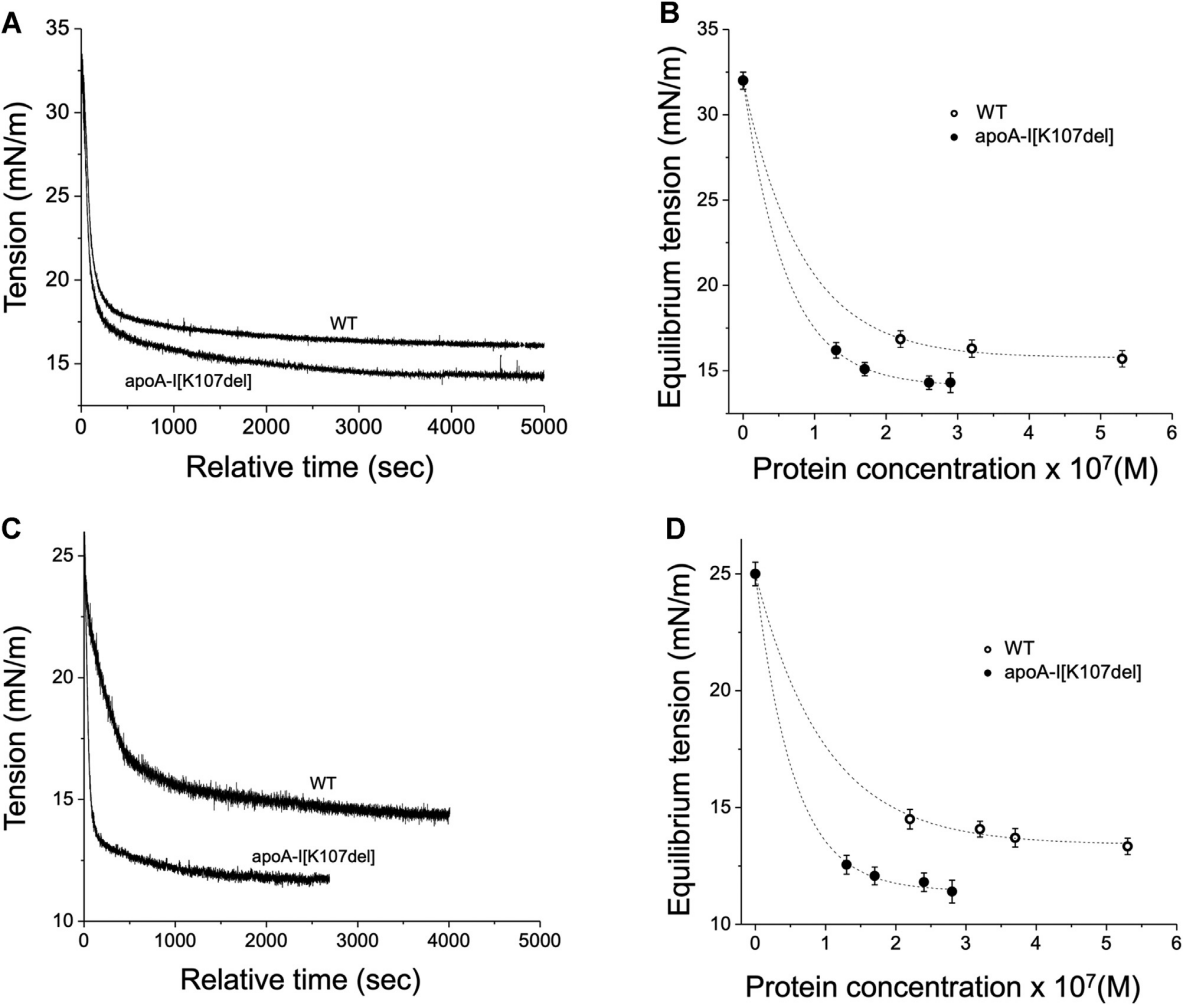
ApoA-I-induced remodeling of various lipid surfaces. A, C: Interfacial tension (γ) changes for TO/W (A) and POPC/TO/W (C) interfaces after adding the proteins to the aqueous phase. The curves are example of the adsorption isotherms (γ vs. time) for WT and apoA-I[K107del] that were added to the aqueous phase at (2.4 ± 0.2) × 10^−7^ M. B, D: Dependence of equilibrium γ on the protein concentration for the TO/W (B) and POPC/TO/W (D) interfaces. Each point represents the average ± S.D. from at least three adsorption isotherms. TO, triolein; W, water.

We showed previously (23) that WT apoA-I binds to TO/W and POPC/TO/W interfaces and decreases interfacial tension γ to equilibrium values, γ_eq_. These decreases in γ correspond to the increases in surface pressure Π calculated as ΔΠ = Π_eq_ – Π_i_. We compared ΔΠ values for WT and apoA-I[K107del] at apolar TO/W and more polar POPC/TO/W interfaces to assess how K107 deletion affects the protein binding to these surfaces. Adsorption isotherms (tension vs. time) were obtained at various protein concentrations in the range 1.3–5.3 × 10^−7^ M. In general, the equilibrium γ depends on protein concentration, with higher protein concentrations resulting in lower γ_eq_ and less time to reach equilibrium. Figure 1A, C show typical pairs of adsorption curves for the proteins at TO/W and POPC/TO/W interfaces, respectively, at protein concentration of (2.4 ± 0.2) × 10^−7^ M. The initial γ of the TO/W interface was 32 ± 0.5 mN/m and that of the POPC/TO/W interface was 25 ± 0.5 mN/m, which corresponds to ᴦ_POPC_ of ∼37% (26). Similar to WT, apoA-I[K107del] lowered γ of both interfaces to reach equilibrium values, but the effect was greater for the mutant than for WT (Fig. 1A, C).

Based on at least three adsorption isotherms for each WT and apoA-I[107del] at (2.4 ± 0.2) × 10^−7^ M at the TO/W interface, WT lowered γ to an equilibrium value of 16.8 ± 0.5 mN/m, while apoA-I[K107del] lowered γ to an equilibrium value of 14.3 ± 0.4 mN/m, The increases in the surface pressure due to adsorption of the proteins, ΔΠ, was higher for apoA-I[K107del] than for WT apoA-I (17.8 ± 0.5 mN/m vs. 15.7 ± 0.5 mN/m, *P* < 0.05), indicating that at the same protein concentration, the mutant remodels the interface to a greater extent. The half time needed to reach equilibrium was calculated from at least three absorption isotherms for each WT and apoA-I[107del] at (2.4 ± 0.2) × 10^−7^ M. The mean half time needed to reach equilibrium at the TO/W interface did not differ significantly between the proteins (85 ± 21 s for apoA-I[K107del] and 99 ± 16 s for WT, *P* > 0.05). Figure 1B shows the tension, γ, of the TO/W surface at equilibrium after adding various concentrations of the proteins. Smaller concentrations of apoA-I[K107del] resulted in larger decreases of surface tension than the higher concentrations of WT apoA-I. For WT at the concentration of 5.3 × 10^−7^ M, γ_eq_ was still higher than γ_eq_ for apoA-I[K107del] at three times lower concentration, 1.7 × 10^−7^ M, indicating that the variant remodels the TO/W interface to greater extent than WT, which is consistent with higher affinity of apoA-I[K107del] to the TO/W interface.

Based on at least three isotherms for each protein at (2.4 ± 0.2) × 10^−7^ M at the POPC/TO/W interface, WT lowered γ to the equilibrium value of (14.5 ± 0.4) mN/ m, while γ_eq_ with apoA-I[K107del] was 11.8 ± 0.4 mN/m. Accordingly, the increase in the surface pressure ΔΠ resulting from the protein adsorption was higher for apoA-I[K107del] than for WT (13.2 ± 0.5 mN/m vs. 10.6 ± 0.5 mN/m *P* < 0.025), indicating that at a similar protein concentration, the variant remodels the POPC/TO/W interface to a greater extent than WT. The half time needed to reach equilibrium calculated from the isotherms was significantly shorter with apoA-I[K107del] than with WT (65 ± 12 s vs. 186 ± 31 s, *P* < 0.01). This difference indicates that in contrast to binding to the TO/W interface, binding to the POPC/TO/W interface was faster for apoA-I[K107del] than for WT. Comparison of γ_eq_ for the POPC/TO/W interface following adsorption of the proteins at various concentrations (Fig. 1D) shows that lower concentrations of apoA-I[K107del] resulted in significantly lower values of γ_eq_ than the much higher concentrations of WT apoA-I. At the relatively high concentration of 5.3 × 10^−7^ M, WT adsorption resulted in γ_eq_ that were still higher than γ_eq_ for apoA-I[K107del] at the three times lower concentration, 1.7 × 10^−7^ M, or even at the four times lower concentration, 1.3 × 10^−7^ M. Taken together, these data indicate that apoA-I[K107del] has a stronger ability than WT to bind to both the TO/W and POPC/TO/W interfaces, and the difference between the proteins is more pronounced at the POPC/TO/W interfaces.

#### Protein penetration into POPC/TO/W interfaces and values of exclusion pressure (Π_EX_)

**Fig. 2:**
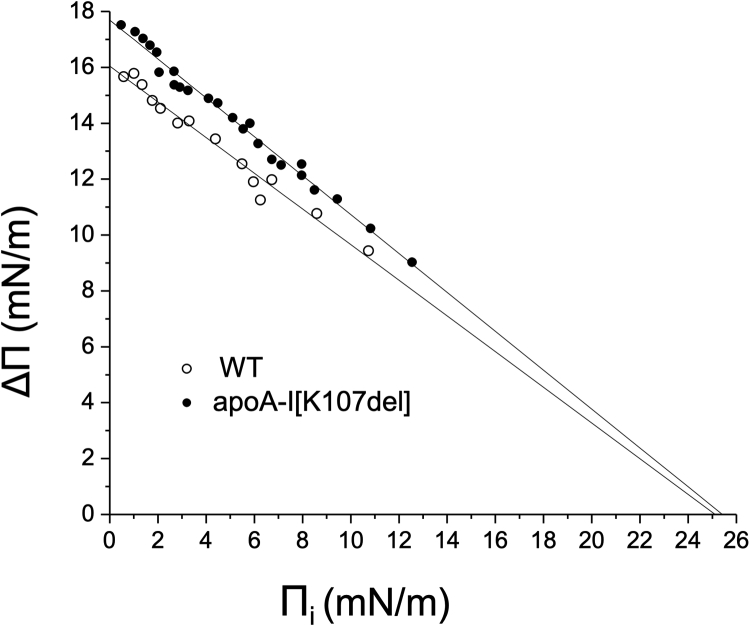
Exclusion pressure Π_EX_ for WT and apoA-I[K107del] at POPC/TO/W interfaces. The initial surface pressure Π_i_ and the corresponding ᴦ_POPC_ of POPC/TO/W interfaces were set as described in Material and Methods. Adsorption of WT or apoA-I[K107del] to the interfaces increased the surface pressure to a different extent, ΔΠ, depending on the initial surface pressure Π_i_. For each protein, changes in surface pressure ΔΠ were plotted against corresponding values of Π_i_, and the data were fitted to linear regressions. X-intercepts of the linear regression at ΔΠ = 0 mN/M represent values of Π_EX_, the exclusion pressure at which a given protein cannot bind POPC/TO/W interfaces. TO, triolein; W, water.

To compare the penetration behavior of WT and apoA-I[K107del] at POPC/TO/W interfaces, the adsorption curves were obtained for each protein at various initial surface pressure Π_i_, corresponding to various surface concentrations of POPC. The values of the increases in the surface pressure, ΔΠ, were plotted against Π_i_, and the data were fitted to linear regression (Fig. 2). Linear regressions for both proteins were significant (R=0.99). Linear regression of the ΔΠ-Π_i_ data for each protein gives Π_EX_ at the x-intercept where ΔΠ = 0 mN/m. Π_EX_ is the surface pressure at which a protein cannot penetrate and bind (i.e., is excluded from) the interface. The values for Π_EX_ were similar for WT and apoA-I[K107del] (25.0–25.5 mN/m). However, the plots show that at each initial surface pressure, binding of apoA-I[K107del] results in larger increases in the surface pressure, indicating that the mutant apoA-I remodels various surfaces of POPC-coated TO droplets to a greater extent and thus, interacts more strongly with the surfaces with various surface concentrations of POPC.

#### Retention pressure (Π_ENV_) on POPC/TO/W interfaces

**Fig. 3:**
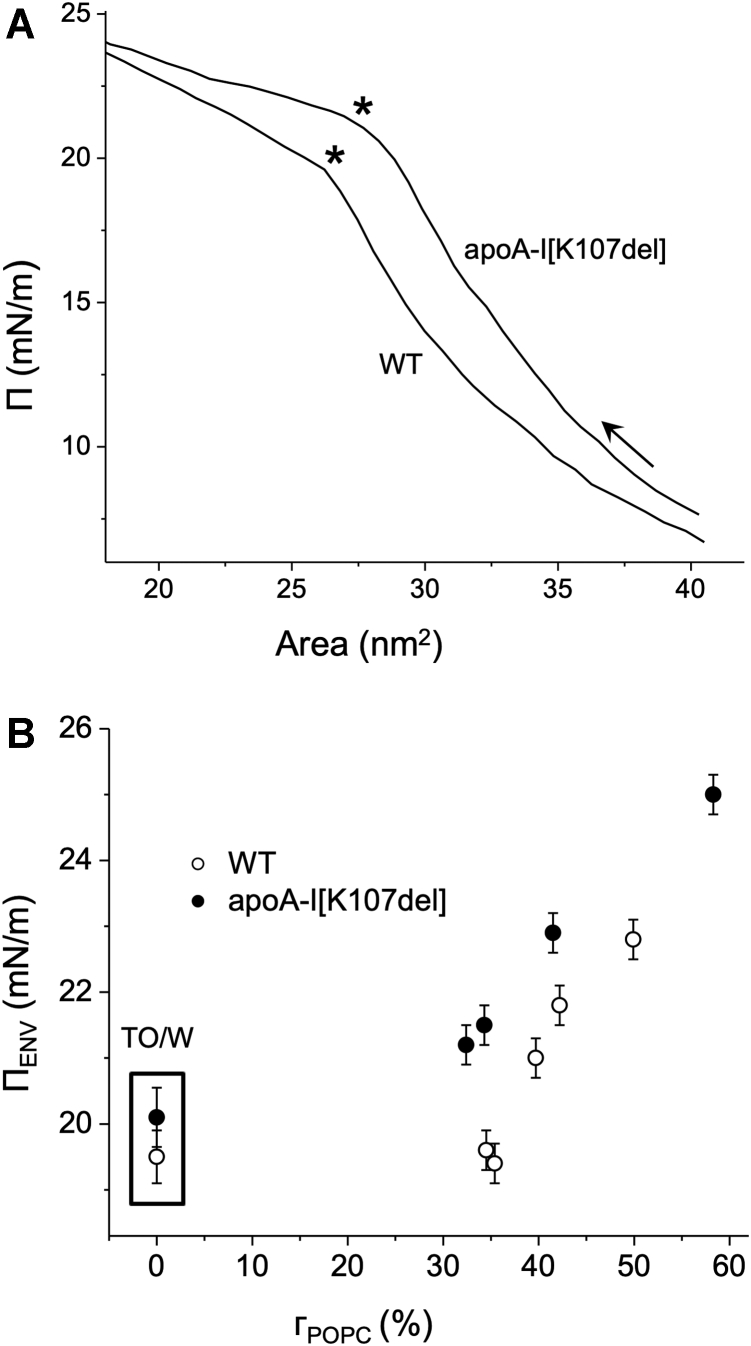
Pressure-area (Π-A) isotherms and retention pressure (Π_ENV_). A: Examples of pressure-area (Π-A) isotherms for WT and apoA-I[K107del] adsorbed to a POPC/TO/W interface of ᴦ_POPC_ = 34.4± 0.1%. WT or apoA-I[K107del], added at 2.7 × 10^−7^ M in the aqueous phase, adsorbed to POPC/TO/W interfaces. Shown here are compression isotherms derived from slow compression following slow expansion of the POPC/TO/W interface with one of the proteins adsorbed to it. The arrow shows the direction of compression. Asterisks mark envelope points (corresponding to A_ENV_ and Π_ENV_), where an abrupt change in slope shows the beginning of ejection of the given protein from the surface upon compression. B: Dependence of Π_ENV_ on POPC surface concentration, ᴦ_POPC,_ for WT and apoA-I[K107del]. Proteins were added at 2.7 × 10^−7^ M in the aqueous phase, adsorbed to the TO/W interface or POPC/TO/W interfaces with varied ᴦ_POPC_. Envelop pressures (Π_ENV_) were determined from Π-A isotherm for each protein and plotted against ᴦ_POPC_. Π_ENV_ values are the mean ± SD, n = 2–3. Π_ENV_ values at TO/W interface (ᴦ_POPC_ = 0%) are outlined by a rectangle. TO, triolein; W, water.

Envelope pressure, Π_ENV_, for WT and apoA-I[K107del] at interfaces with varied POPC surface concentration, ᴦ_POPC_, was determined to compare differences in the protein retention over a range of lipid/water interfaces. To obtain the values of Π_ENV_, compression Π-A isotherms were recorded for POPC/TO/W surfaces with various POPC surface concentrations. Figure 3A shows examples of the Π-A isotherms for WT and apoA-I[K107del] adsorbed to POPC/TO/W interface with ᴦ_POPC_ = 34.4 ± 0.1%. The data were generated from γ and surface area profiles when the POPC-coated TO drop with a protein adsorbed into the surface was slowly expanded and then compressed. The arrow marks the direction of compression. The asterisk (∗) marks a point of change of the isotherm slope and corresponds to the envelope point, that is the surface area and pressure (Π_ENV_) at which the protein begins to be ejected from the surface on compression. The higher value of the Π_ENV_ for the mutant (21.5 ± 0.5 mN/m vs. 19.6 ± 0.5 mN/m for WT, *P* < 0.05) indicates that compared to WT, apoA-I[K107del] begins to be ejected from the surface at higher surface pressure suggesting better retention of the variant on the POPC/TO surface. The Π-A isotherms for both proteins were obtained for various initial drop volumes corresponding to the various initial surface pressure and ᴦ_POPC_. Values of Π_ENV_ determined from the isotherms for various ᴦ_POPC_ are shown in Fig. 3B. The values of Π_ENV_ at ᴦ_POPC_ = 0% (marked by a box) were determined from Π-A isotherms for the TO drop without POPC coating. Retention pressure for the two proteins did not differ significantly on the surface of the TO drop without POPC. In contrast, for POPC-coated TO drops, at each POPC surface concentration studied, apoA-I[K107del] was ejected from the surfaces at higher surface pressures than WT, indicating stronger retention of the variant on the POPC/TO/W interfaces.

### Effect of K107 deletion on cholesterol efflux and nHDL biogenesis

Although the capacity of apoA-I[K107del] to promote ABCA1-mediated cholesterol efflux was reported by others using different cells (17, 27, 28), we confirmed this process in J774 cells used in this study. We therefore analyzed the capacity of the mutant apoA-I[K107del] to promote cholesterol efflux over a 6- and 24-h incubation period and compared the data with WT apoA-I. We chose these time points to investigate potential differences that may be occurring either during short and/or long incubation times (31).

**Fig. 4:**
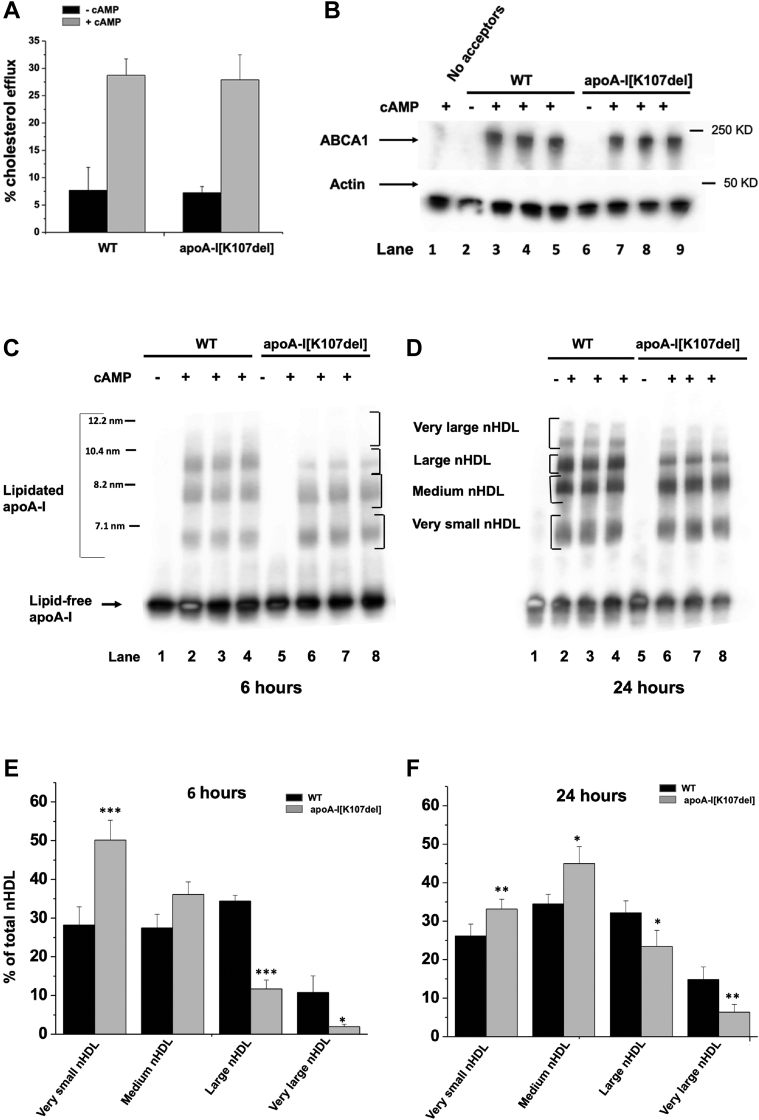
ABCA1-mediated cholesterol efflux and nHDL formation. A: Cholesterol efflux. Cells prelabeled with BODIPY-cholesterol were incubated with either WT or apoA-I[K107del] in media containing (gray bars) or not containing cAMP (black bars) for 24 h. Cholesterol efflux was determined by measuring net fluorescence in media and expressed as a percentage of total fluorescence in cells plus media. Bars represent means ± SD (-cAMP, n = 3; +cAMP, n = 4). B: ABCA1 levels. Cells incubated with either WT or apoA-I[K107del] as described in Panel A were analyzed by Western blotting. Membranes were probed with antibodies to ABCA1 or to pan actin (loading control). Lane 1 is from cells incubated without protein acceptors. C, D: Immunoblots showing apoA-I. Media harvested following a 6- (C) or 24-h (D) incubation with acceptors were analyzed by nondenaturing gradient (4%–15%) PAGE followed by immunoblotting. Immunoblots were probed with antibodies to apoA-I. Lanes 1–4 and 5–8 represent media derived from cells incubated with WT or lapoA-I[K107del], respectively. Lanes 1 and 5 represent media from cells incubated in the absence of cAMP. Lanes 2–4 and 6–8 represent triplicate media samples derived from cells incubated with cAMP. E, F: Lipidated apoA-I bands labeled as very small, medium, large, and very large nHDL depicted in panels C and D were quantified and expressed as % of total nHDL formed after 6 and 24 h, respectively. Bars represent means ± SE (n = 5). ∗*P* < 0.05; ∗∗*P* < 0.01; ∗∗∗*P* < 0.001. nHDL, nascent HDL.

Figure 4A shows that, as expected, following a 24-h incubation, the efflux promoted both by the WT and apoA-I[K107del] was relatively low from cells not incubated with cAMP and corresponded to 7.7 ± 4.2 and 7.3 ± 1.1%, respectively. Indeed, these cells have very low or undetectable level of ABCA1 (Fig. 4B, lanes 2 and 6). However, both apoA-I[K107del] and WT promoted higher efflux (Fig. 4A) from cells treated with cAMP that upregulated ABCA1 (Fig. 4B, lanes 3–5 and 7–9). Percent cholesterol efflux promoted by apoA-I[K107del] was similar to that promoted by WT and corresponded to 27.9 ± 4.6 and 28.7 ± 2.8%, respectively. Thus, the majority of the efflux was ABCA1-mediated. Furthermore, efflux promoted by apoA-I[K107del] after 6-h incubation was also similar to that of WT and corresponded to ∼ 60% of the efflux measured after 24-h incubation (data not shown).

The level of ABCA1 in cells incubated with apoA-I[K107del] for 24 h was similar to that in cells incubated with WT (as determined in two experiments run in triplicates and normalized to actin level). In addition, cells treated with cAMP that were incubated in media not containing protein acceptors expressed a very low level of ABCA1 (Fig. 4B, lane 1). Thus, similar to previous reports (17, 28, 29), K107 deletion does not impair the ability of the mutant to promote cholesterol efflux as efficiently as the WT.

Given that efflux promoted by the apoA-I[K107del] mutant was similar to that promoted by the WT, it was important to determine whether the mutant can bind the effluxed lipids as efficiently as the WT to form nHDL particles. To that end, we analyzed efflux media collected from cells after 6- and 24- h incubation by Western blotting. Figure 4C, D show representative immunoblots (probed with antibodies to apoA-I) of efflux media harvested after a 6- and 24-h incubation, respectively. As shown in these immunoblots, there was a major band of apoA-I in media derived from cells incubated without cAMP representing lipid-free apoA-I (lanes 1 and 5) that was not lipidated due to low or undetectable level of ABCA1 (Fig. 4B, lanes 2 and 6) resulting in minimal efflux (Fig. 4A). When WT or apoA-I[K107del] were added to cells in the presence of cAMP to upregulate ABCA1 (Fig. 4B, lanes 3–5 and lanes 7–9), they became lipidated and formed nHDL particles as early as 6 h as indicated by the presence of multiple bands representing a heterogenous population of presumably discoidal nHDL particles ranging from ∼6.8 to 12.2 nm diameter (Fig. 4C, D, lanes 2–4, and lanes 6–8, respectively). However, quantification of all bands representing nHDL showed that the overall capacity to form particles with apoA-I[K107del] was reduced to 92 ± 6% (*P* < 0.05) and 93 ± 7% (*P* = 0.07) of that for WT following 6- and 24-h incubation, respectively. The reduction in the ability of apoA-I[K107del] to bind the effluxed lipids was primarily due to its reduced capacity to form nHDL particles larger than 8.5 nm diameter (compare lanes 6–8 to lanes 2–4 of Fig. 4C, D) representing large and very large nHDL particle (35). After 6- and 24-h incubation, the relative abundance of large and very large nHDL particles formed by the mutant was significantly lower than those formed by the WT (Fig. 4E, F). Large nHDL particles formed by the mutant after 6- and 24-h incubation accounted for 11.7 ± 2.3% (*P* < 0.001) and 23.4 ± 4.2 (*P* < 0.02), of total nHDL, respectively. Very large nHDL particles formed by apoA-I[K107del] after 6- and 24- h incubation accounted for 1.9 ± 0.6 (*P* < 0.05) and 6.4 ± 2% (*P* < 0.005) of total nHDL, respectively. For comparison, the relative abundance of large nHDL particles formed by the WT after 6- and 24-h incubation was 34.1 ± 1.5 and 32.2 ± 3.1%, respectively, and the relative abundance of very large nHDL particles was 10.8 ± 0.8 and 14.8 ± 5.7%, respectively.

It is notable that continued incubation from 6 to 24 h led to a small increase in the relative abundance of very large nHDL particles formed by the WT from 10.8% to 14.8% and a significant (*P* < 0.05) increase by the mutant from 1.9% to 6.4% of total nHDL, respectively. On the other hand, while the abundance of large nHDL particles formed by the WT did not change, this population of nHDL particles formed by the mutant increased significantly (*P* < 0.05) from 11.7% to 23.4% of total nHDL (Fig. 4E, F, respectively). Nevertheless, the overall abundance of combined large and very large nHDL particles formed by the mutant both after 6 and 24 h was significantly lower than those formed by WT (*P* < 0.0001 and *P* < 0.001, respectively).

Overall, the relative abundance of large plus very large nHDL particles formed by apoA-I[K107del] after 6- and 24- h incubation was significantly reduced to 30.4 ± 13% (*P* < 0.0001) and 63.4 ± 3% (*P* < 0.002) of the relative level of these particles formed by the WT, respectively (supplemental Fig. S1).

These findings indicate that the mutant apoA-I[K107del] formed large and very large nHDL particles either more slowly or of lower stability than WT and thus was unable to attain WT levels even after a 24-h incubation.

Since we previously demonstrated that nHDL particles formed by apoA-I following efflux from transfected HEK 293 cells contained the ganglioside GM1 (31) it was of interest to determine whether a) nHDL particles formed by apoA-I incubated with J774 cells also contain GM1 and whether the mutant has the capacity to bind GM1as efficiently as WT and b) to confirm that the lipid distribution (represented by GM1) is similar to the distribution of apoA-I. To determine the content of GM1 in nHDL particles, we probed the membranes with cholera toxin subunit B which specifically binds to GM1 (32). The representative ligand blots in supplemental Fig. S2A, B show that the distribution of GM1 in media derived from both WT and apoA-I[K107del] after 6- and 24-h incubation parallels the distribution of lipidated apoA-I (Fig. 4C, D, lanes 2–4 and 6–8). Importantly, all nHDL particles formed by WT and apoA-I[K107del] contained GM1 (lanes 2–4 and 6–8). No GM1 was detected in media derived from cells that were not incubated with cAMP (supplemental Fig. S2A, B, lanes 1 and 5). Large plus very large (e.g., >8.5 nm-diameter) nHDL particles formed by the mutant contained 22 ± 6% and 25 ± 6% of total GM1 in nHDL following 6- and 24-h incubation, respectively, which were significantly lower than the relative level of these populations of nHDL particles formed by the WT and corresponded to 49 ± 15% and 61± 9%, of WT, respectively (*P* < 0.01) (supplemental Fig. S2C). Overall, deletion of K107 did not impair the ability of apoA-I to bind GM1 but due to diminished biogenesis of large and very large nHDL particles, there was proportionally less GM1.

Together, these findings suggest that deletion of K107 impairs the ability of apoA-I to bind the effluxed lipids and form nHDL particles as efficiently as the WT and may impair the protein ability to bind enough lipid to form large and very large nHDL particles resulting in the biogenesis of primarily smaller nHDL particles. Such changes are likely to have a profound impact in vivo.

### Effect of K107 deletion on sizes of DMPC-apoA-I complexes and α-helicity of apoA-I

**Fig. 5:**
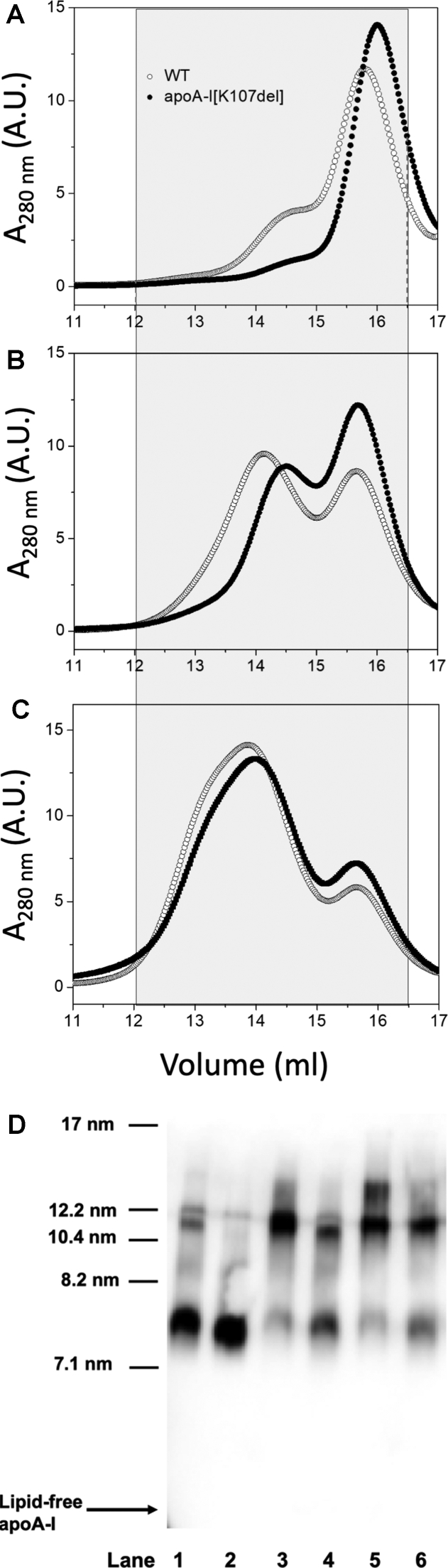
Size-exclusion chromatography of DMPC-apoA-I complexes. A–C: Gel filtration elution profile of DMPC-apoA-I complexes. WT and apoA-I[K107del] were incubated with DMPC suspension for 66 h at 24°C at DMPC:apoA-I weight ratio of 2 (A), 4 (B), or 8 (C). The mixtures were then eluted through Superose 6 HP 10/30 column equilibrated with TBS. Fractions containing DMPC-apoA-I complexes (gray-shaded areas) were pooled for analysis. Profiles for WT and apoA-I[K107del] shown by empty circles and filled circles, correspondingly. D: Representative immunoblot of DMPC-apoA-I complexes. Pooled fractions containing DMPC-apoA-I complexes were analyzed by nondenaturing gradient (4%–15%) PAGE followed by immunoblotting with antibodies to apoA-I. Lanes 1, 3, and 5: WT-containing complexes separated by SEC for DMPC:apoA-I ratios of 2, 4, and 8, correspondingly. Lanes 2, 4, and 6: apoA-I[K107del]-containing complexes separated by SEC for DMPC:apoA-I ratios of 2, 4, and 8, correspondingly. Position of lipid-free apoA-I on the blot is indicated by the arrow. DMPC, 1,2-dimyristoylphosphatidylcholine; SEC, size-exclusion chromatography.

We aimed to investigate the properties of the apoA-I[K107del] that may contribute to the impaired ability of the protein to recruit lipids and form large and very large nHDL particles. To this end, we incubated apoA-I with DMPC at increasing lipid:protein ratios and studied the resultant DMPC-apoA-I complexes, which are commonly used as simple models of nHDL. Figure 5, A–C, shows SEC elution profiles of the resultant DMPC-apoA-I complexes formed at DMPC; apoA-I weight ratio of 2, 4, and 8, corespondingly. Both lipid-free WT and apoA-I[K107del] incubated without DMPC were eluted from the column between 17 and 19 ml, with a peak volume of 18 ml (not shown). Therefore, the fractions between 12 and 16.5 ml collected for analysis contained DMPC-apoA-I complexes with no lipid-free apoA-I. For each DMPC:apoA-I ratio, the elution profiles for apoA-I[K107del]-containing complexes were shifted to larger elution volumes compared to those for WT-containing complexes, indicating overall smaller hydrodynamic diameters of the particles formed by the mutant. For each DMPC:apoA-I ratio, the elution profiles show two major populations of particle with different hydrodynamic diameters, with the apparent peak heights corresponding to the smaller particle being greater for the mutant than for WT.

Accordingly, the representative immunoblot of the pooled fractions (Fig. 5D) for each DMPC:apoA-I ratio shows a band corresponding to smaller particles (with Stokes diameters between 7.4 – 7.8 nm) and one band or two bands corresponding to larger particles (with Stokes diameters between 10.5 - 14 nm). Thus, for each DMPC:apoA-I ratio, apoA-I[K107del]-containing complexes show increased relative proportion of smaller particles and reduced abundance of larger particles. Furthermore, although increasing DMPC:apoA-I ratio from 2 to 8 led to increased relative abundance of larger particles for both WT and the mutant, WT formed more of the larger particles. Remarkably, increasing DMPC:apoA-I ratio up to 8 led to the formation by WT of a distinct population of particles with diameters of ∼14 nm, in addition to the particles with ∼10.5 nm diameter. The mutant, on the other hand, was unable to form this population of the largest particles (compare lane 5 to lane 6 in Fig. 5D). Notably, the blot showed no presence of lipid-free apoA-I in any of the samples of WT- or apoA-I[K107del]-containing complexes separated by SEC. Overall, our findings are consistent with the hampered ability of apoA-I[K107del] variant to form larger discoidal complexes with DMPC.

Our findings agree with a significant shift of DMPC-apoA-I complexes toward smaller sizes as a result of the K107del mutation that was found by Huang *et al.* (17) for the complexes formed by sodium cholate dialysis at DMPC:apoA-I weight ratio of 3.9 (molar ratio of 150:1). For a close DMPC:apoA-I weight ratio of 3.8 (molar ratio of 145:1), Ludovico *et al.* (33) found no significant effect of the K107del mutation on sizes of DMPC-apoA-I complexes based on the quantification of gradient nondenaturing gels. Interestingly that when DMPC-apoA-I complexes were formed by sodium cholate dialysis (17), deletion of K107 resulted in an additional population of smaller particles that were not formed by WT and in a lack of a minor population of the largest particles that were formed by WT. In our studies, both WT and apoA-I[K107del] were able to form a population of smaller particles. Thus, the effect of K107 deletion was not quite the same for DMPC-apoA-I particles prepared by sodium cholate dialysis and spontaneously reconstituted DMPC-apoA-I particles. This agrees with the recent findings by Bedi *et al.* (36), suggesting that structural requirements for apoA-I to form complexes with phospholipids spontaneously may be different from those needed for the detergent-assisted formation of the complexes. Given that K107del mutation affects the protein conformation and stability (15, 16), the altered structural properties of apoA-I[K107del] apparently translated into slightly different effects for DMPC-apoA-I complexes formed spontaneously or by cholate dialysis method. Importantly, regardless of the method of the particle formation, the K107del mutation resulted in the shifts of DMPC-apoA-I particles to smaller sizes.

**Fig. 6:**
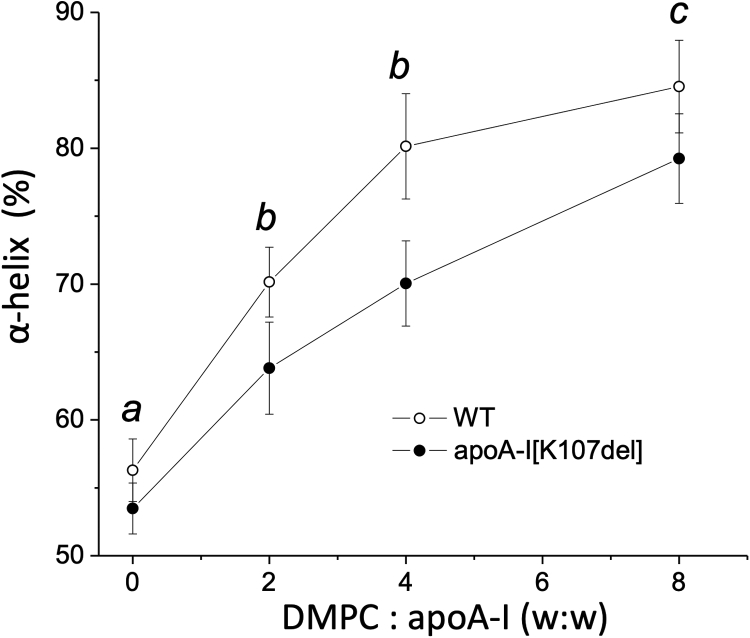
The α-helical content of apoA-I in DMPC-apoA-I complexes. DMPC-apoA-I complexes obtained by spontaneous reconstitution by incubating WT and apoA-I[K107del] with DMPC at various lipid:protein ratios and separated by SEC, as shown in Figure 5, A–C, were analyzed by far-UV CD. The α-helical content of apoA-I in the DMPC-apoA-I complexes is plotted against DMPC:apoA-I ratios in the incubation mixtures. Data are means ± SD, n ≥3. Letters above the data points indicate significance of the difference between the values for apoA-I[K107del] and WT by unpaired *t*-test, *a:* not significant, *b: P* < 0.01, *c*: *P* < 0.05. DMPC, 1,2-dimyristoylphosphatidylcholine; SEC, size-exclusion chromatography.

It is well documented that the interaction of apoA-I with phospholipids to form discoidal HDL complexes is driven by an increase in amphipathic helical content in the protein on lipid interaction. To test the effect of the K107 deletion on the ability of apoA-I to increase α-helical structure on binding to DMPC, we determined the α-helical content of apoA-I in the DMPC-apoA-I complexes formed at increasing DMPC:apoA-I ratios. For the CD analysis, we used the same pooled fractions collected from SEC that we used for the immunoblot analysis, that is complexes formed at DMPC:apoA-I ratios of 2, 4, and 8 (Fig. 5, A–C, correspondingly). The dependence of apoA-I α-helix on DMPC:apoA-I ratio is shown in Fig. 6. Consistent with our earlier findings (15), the K107del mutation did not change significantly the α-helix content of lipid-free apoA-I. However, the mutation resulted in the reduced α-helical content of apoA-I in DMPC-bound state at each DMPC:apoA-I ratio. Consistent with the SEC data, the largest effect of the K107 deletion on the protein α-helical content (∼10% decrease) was observed at DMPC:apoA-I ratio of 4. This reduction in the α-helical content, as a result of the K107del mutation, corresponds to unfolding of ∼ 24-residue segment that is roughly one sequence repeat in apoA-I. At DMPC:apoA-I ratios of 2 and 8, the K107 deletion resulted in smaller but statistically significant reductions in the protein α-helical content, 6%–7% and 5%–6%, correspondingly. These reductions in the α-helical content resulted from the deletion of K107 correspond to unfolding of segments that are smaller than one sequence repeat. Thus, CD analysis of DMPC-apoA-I complexes indicate that deletion of K107 impedes the ability of apoA-I to increase α-helical structure on binding to the phospholipids.

### Effect of K107 deletion on the α-helical content of apoA-I in the presence of TFE

**Fig. 7:**
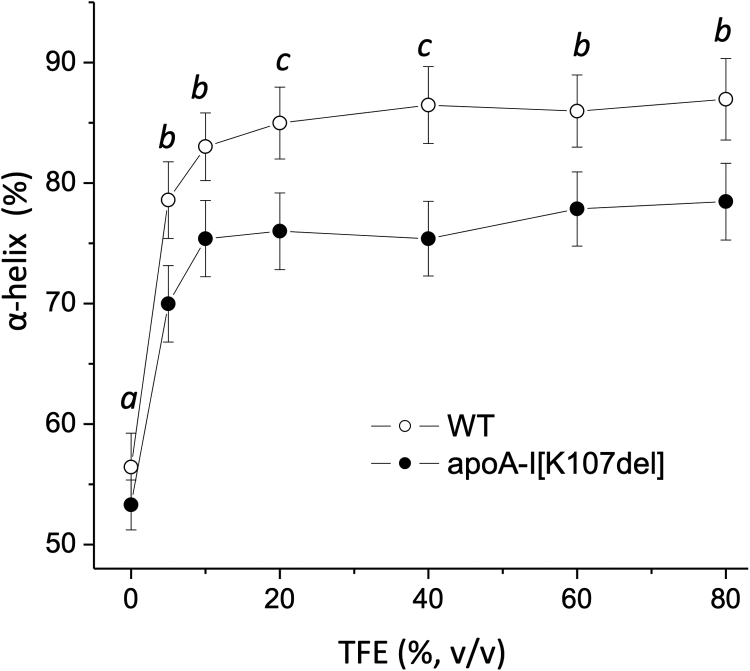
The α-helical content of apoA-I in the presence of TFE. WT or apoA-I[K107del] were incubated in 10 mM phosphate buffer for 1 h at 4°C with various concentrations of TFE, and the α-helical content was determined from far-UV CD spectra. Data are means ± SD, n = 3. Letters above the data points indicate significance of the difference between the values for apoA-I[K107del] and WT by unpaired *t*-test, *a:* not significant, *b: P* < 0.05, *c: P* < 0.01. TFE, trifluoroethanol.

To test if K107del affects the ability of apoA-I to fold additional α-helices in other environments that stimulate formation of α-helical structure, we determined the α-helical content of WT and apoA-I[K107del] in the presence of various concentrations of the helical structure inducer TFE (Fig. 7). For both proteins, the α-helical content progressively increased when TFE concentration rose from 0% to 10%–20%, and further increase in the TFE concentration did not result in significant changes of the protein α-helicity. However, while the α-helical content of the two proteins in the aqueous buffer (TFE = 0%) did not differ significantly, apoA-I[K107del] had a significantly lower α-helical content than WT in the presence of the various concentrations of TFE. The maximum α-helicity induced by TFE was on average 10% lower for apoA-I[K107del] compared to WT, again corresponding to the number of residues in one sequence repeat. Thus, K107del mutation impairs the ability of apoA-I to increase α-helical structure in the presence of TFE.

## Discussion

Despite many published studies on human apoA-I[K107del] variant that is associated with low HDL-C levels (10, 11, 12, 18) and premature CVD (13, 18), its properties are not fully understood. Some conclusions regarding the effects of K107 deletion on apoA-I properties, such as LCAT activation (17, 18, 37, 38), DMPC solubilization rate (15, 17, 33), or aggregation in solution (16, 39), are inconsistent. One of the unusual findings about apoA-I[K107del] is that it is not associated with low plasma apoA-I concentrations, despite being associated with low HDL-C (10, 11). This phenotype contrasts with that of most human apoA-I mutations that are associated with either both reduced plasma HDL-C and reduced apoA-I levels (8, 10, 40, 41, 42) or elevated HDL-C along with elevated plasma apoA-I levels (40). In this work, we revealed important properties of apoA-I[K107del] that may contribute to its unusual phenotype.

Our earlier studies on binding of apoA-I to synthetic TG-rich emulsion particles (15) suggested that the K107del mutation may enhance binding of apoA-I to large TG-rich lipoproteins in vivo. However, no other published experimental results on this variant or data on patients carrying this mutation that would directly support this hypothesis were known. In the current study, we studied for the first time lipid surface behavior of apoA-I[K107del] and revealed properties of the variant that can drive its increase affinity to large TG-rich lipoproteins. We found that deletion of K107 led to increases in both the extent and the rate of adsorption of apoA-I to the surfaces of POPC-coated TO drops and increased retention of apoA-I on the surfaces. The differences between apoA-I[K107del] and WT in adsorption (Fig. 1) and especially desorption (Fig. 3B) behaviors were more pronounced for the more polar POPC-TO surfaces than for the surface of a TO drop without POPC. The higher Π_ENV_ values for apoA-I[K107del] on the surface of POPC-coated TO drops (Fig. 3B) imply that in vivo, during lipoprotein remodeling and inferred lipoprotein surface pressure changes, apoA-I[K107del] remains on the surfaces of TG-rich particles up to higher pressures than WT. The larger surface pressure changes that resulted from adsorption of apoA-I[K107del] to the surfaces of POPC-coated TO drop (Fig. 2) indicate that apoA-I[K107del] remodels the lipid surfaces to a greater extent than WT, suggesting stronger interactions of the variant with the surface lipids of TG-rich lipoproteins. Taken together, these observations imply that enhanced binding of apoA-I[K107del] to the phospholipid surfaces of TG-rich lipoproteins and its better retention on these surfaces may lead to an increased content of apoA-I[K107del] on TG-rich lipoproteins in vivo.

It has been shown that compared to WT, apoA-I[K107del] has a less stable and more loosely folded lipid-free conformation with greater exposure of hydrophobic surfaces (15, 16). It has been proposed that lipid-free apoA-I forms a four-segment bundle that opens when the protein binds to lipids or lipoprotein lipid surfaces [(1) and references sited therein]. In our earlier studies, we suggested that the K107 deletion results in helical registry shift and ensuing disruption of salt bridges, both intra-helical (K107-E110 and K107-E111) and interhelical (E111-H155 and E111-R151) that leads to destabilization of the N-terminal helical bundle structure and greater exposure of hydrophobic surfaces in lipid-free apoA-I (15). These characteristics are consistent with the position and interactions of K107 in the crystal structure of the [1–184] apoA-I dimer, as well as the proposed “domain swapped” monomer (43) and the consensus structure of lipid-free apoA-I (44). Specifically, in all three structural models, K107 forms a strong intra-helical salt bridge with E110 and E111 located in helix 4 that is a part of the N-terminal helix bundle. In addition, as a consequence of the loss of the K107/E111 interaction, interhelical salt bridges (E111-H155 and E111-R151) that stabilize the interaction between helix 4 and helix 6 in the bundle are also likely to be perturbed. Thus, deletion of K107 is expected to shift the helical registry, disrupts these salt bridge interactions, and thereby leads to destabilization of the helix bundle and exposure of the hydrophobic core of the bundle. The lower stability and more losely folded conformation leading to exposed hydrophobic surfaces would provide flexibility and adaptability for conformational changes that are required for protein binding to the surface of large TG-rich lipoprotein particles (15). These processes represent the structural basis of the enhanced binding of apoA-I[K107del] to the surfaces of TG-rich lipoproteins (or lipid drops in the drop tensiometry experiments). It should be noted that the general structural organization of apoA-I on TG-rich particles must be different from that on discoidal or spherical HDL particles. Given that sizes of TG-rich particles are much larger than HDL (50–100 nm for VLDL vs. 7–12 nm for HDL), and the relative content of apoA-I on TG-rich particles is expected to be immensely lower than on HDL, apoA-I is not able to form cage-like structures on TG-rich particles. Furthermore, the lipid drops used in our drop-tensiometry experiments are much larger than VLDL.

Remarkably, animal studies showed that when human WT or variant forms of apoA-I were expressed in apoA-I -/- mice, apoA-I mutations that led to similar conformational changes (less stable and more loose folding with greater exposure of hydrophobic surfaces) resulted in a noticeable portion of apoA-I detected in plasma TG-rich lipoproteins of the animals [(45, 46), ref. (47) and references therein]. In one study (46), up to 40% of an apoA-I variant with the similar conformational characteristics were detected in plasma TG-rich lipoproteins.

Another factor contributing to low HDL-C along with normal apoA-I levels in plasma of individuals with the K107del mutation may relate to the smaller sizes and reduced cholesterol content of their HDL particles, first reported by Tilly-Kiesi *et al.* (18). As apoA-I is an activator of LCAT that promotes enlargement of HDL in the circulation, several studies investigated if the K107 deletion affected the ability of apoA-I to activate LCAT. The LCAT activation ability of apoA-I[K107del] isolated from heterozygous individuals was found to be either lower than (37, 38) or similar to (18) that of WT apoA-I isolated from the same individuals. Studies of recombinant pro-apoA-I also did not find any effect of K107 deletion on the protein’s LCAT activation ability (17). Thus, it seems unclear if the reduced HDL size associated with apoA-I[K107del] may relate to impaired cholesterol esterification by LCAT. We posited that structural changes in apoA-I[K107del] may modify the initial step of HDL biogenesis. To test this hypothesis, we investigated the effect of K107del on ABCA1-mediated cholesterol efflux and sizes of nHDL.

We found no effect of K107 deletion on apoA-I ability to promote ABCA1-mediated cholesterol efflux from J774 cells, in agreement with earlier reports (17, 27, 28) that found no effect of K107del on net cholesterol efflux from fibroblasts, CHO cells, and murine adipocytes. As apoA-I is believed to directly interact with ABCA1 on cell membranes (31, 48), these data suggest that deletion of K107 does not affect binding of apoA-I to ABCA1. Similar levels of ABCA1 in the presence of WT or apoA-I[K107del] (Fig. 4B) are also consistent with similar interactions of both proteins with ABCA1. It is known that apolipoproteins stabilize ABCA1 in cells by protecting it from proteases (49). Interestingly, Vedhachalam *et al.* (50) proposed that hydrophobicity of the C-terminal domain of apoA-I is critical for effective ABCA1-mediated cholesterol efflux, while destabilization of the N-terminal bundle may increase the effectiveness, and a cooperation of the N-terminal and C-terminal domains enhance cholesterol efflux to apoA-I via ABCA1. It is possible that the intact C-terminal domain and cooperation between the N- and C-terminal domains preserved in the apoA-I[K107del] variant override the potentially enhanced ability of the destabilized N-terminal bundle to promote cholesterol efflux. Our observations for the K107del mutation are similar to those reported for the L38G/K40G mutation of apoA-I that we studied previously (31). In both cases, the mutation did not result in changes in ABCA1-mediated cholesterol efflux from cells but had a marked effect on nHDL biogenesis, supporting our suggestion that efflux and nHDL formation are uncoupled processes. The L38G/K40G mutation, designed to destabilize a hinge region but have little effect on the helical backbone, resulted in a significantly enhanced ability to form nHDL, which suggests that a destabilized N-terminal bundle facilitates nHDL formation. In contrast, despite no effect of on ABCA1-mediated cholesterol efflux, the K107 deletion mutation modulated the size distribution of nHDL particles, resulting in a reduced abundance of larger particles and an increased proportion of smaller particles. While the relative level of large plus very large nHDL particles formed by the mutant was higher after a 24-h incubation compared to 6-h incubation, this population was still significantly lower than for the WT. Furthermore, in contrast to WT, apoA-I[K107del] was not able to form the largest nHDL (with diameter >10 nm) (Fig. 4C, D).

It was demonstrated that the relative amount of cholesterol varies with the size of HDL particles. Mendivil *et al.* (35) showed that the ratio of cholesterol to apoA-I increases with HDL size in humans. Similarly, Liu *et al.* (51) reported that the cholesterol:phospholipid ratio in the larger nHDL particles formed by apoA-I following incubation with J774 cells is three times higher than in the smaller particles. Therefore, in our system, even after the prolonged incubation of 24 h, the 36% reduction in the relative abundance of large plus very large nHDL translates into a much larger reduction in total HDL-C. Since very small and medium nHDL particles are enriched in phospholipids, their relative increase (Fig. 4E, F) cannot compensate for the reduction in total cholesterol, thus contributing to the factors that may lead to low HDL-C in patients carrying K107del mutation in A-I (18, 52).

Taken together, our findings show that apoA-I[K107del] exerts the similar effect on the size of discoidal HDL particles, whether they are nHDL formed in cells by ABCA1 reaction or DMPC-apoA-I complexes created by lipid solubilization. The structural limitations that prevent formation by apoA-I[K107del] of larger discoidal particles in the experimental conditions may prevent formation of larger discoidal and spherical HDL in plasma of patients carrying this mutation. Indeed, it was shown that plasma of patients heterozygous for apoA-I[K107del] was lacking larger HDL_2_ subfractions and contained mostly smaller HDL_3_ subfractions (18), thereby increasing the patients' risk for atherosclerosis and CVD.

In contrast to apoA-I binding to large TG-rich particles, an important structural self-association of apoA-I molecules is required when apoA-I binds to HDL or solubilizes lipids on the surface of ABCA1-expressing cells to form discoidal nHDL or solubilizes phospholipid vesicles to form discoidal phospholipid-apoA-I complexes. ApoA-I must dimerize to form “double-belt” structures around the lipid bilayer of the nHDL discs. The general structural organization of apoA-I is thought to be similar overall on discoidal and spherical HDL particles, with the “double-belt” being the fundamental organizational motif for apoA-I (53, 54). In discoidal particles, the “double-belt”, comprised of two antiparallel apoA-I molecules, wraps around the edge of the discs (55), and in spherical particles, 3 to 5 “double-belted” apoA-I molecules form a symmetrical cage-like structure around the spheres (53, 54). Some data suggest that the presence of a third apoA-I molecule in a ‘hairpin” conformation cannot be ruled out for discs with diameters exceeding 10 nm (1, 56, 57).

It is well documented that the sequence of apoA-I contains ten, potentially amphipathic helical repeats in the exon 4 encoded region (residues 44–243) that form this double-belt together with the N-terminal (residues 1–43) domain. In the “double-belt” arrangement, the polar surface of apoA-I amphipathic helices faces the aqueous environment, while the nonpolar face of the amphipathic helices interacts with the hydrophobic regions of phospholipids, shielding them from exposure to water and thereby stabilizing the HDL particle (55). Segments of the apoA-I molecules forming these “double-belts” may have loop or unstructured character in some of the repeat segments of the apoA-I molecules that are displaced from the particle surface or twisted to modulate particle diameter in response to various amount of lipid cargo (53, 56, 57, 58). Thus, the formation of the double-belt to stabilize smaller-sized discoidal HDL particles does not require all ten repeat segments to form helical conformation. As particles increase in size, unstructured segments of the sequence repeats are driven to form in-register amphipathic helical structure to expand the antiparallel double-belt and stabilize the increased perimeter of the particle. The α-helical content of apoA-I on both discoidal and spherical synthetic HDL is known to increase when the lipid:apoA-I ratio and diameter of the particles increases (59). This observation is supported by hydrogen exchange and mass spectroscopy analysis of discoidal particles (58). Our analysis of the α-helical content of WT and apoA-I[K107del] with increasing concentrations of DMPC or TFE reveals that in both environments, apoA-I[K107del] was not able to increase α-helical structure as much as WT (Figs. 6 and 7).

Most importantly, stabilizing interactions between the two apoA-I molecules forming the double-belt are achieved through extensive salt bridges between the antiparallel helices. As discussed earlier (15), in addition to residue K107 forming an important stabilizing intra-helical salt-bridge (K107-E111) in the structure of WT apoA-I, K107 together with several other residues is involved in critical salt-bridge networks that stabilize the helix 4-helix 6 interaction of the apposed molecules in the double-belt. Thus, overall, deletion of K107, together with the resulting disruption of the registry of the polar and apolar helical faces, disrupts and destabilizes this region with consequent disruption of important interhelical stabilizing interactions that form the double-belt. The hampered ability of apoA-I[K107del] to form correctly structured α-helix at higher lipid:protein ratios impairs the variant’s ability to form an appropriately structured and stabilized “double-belt” around larger HDL, both discoidal and perhaps spherical HDL. In small HDL particles, the helix4-helix 6 region may adopt a loop/unstructured conformation instead of helical structure due to lower lipid load. Thus, the apoA-I[K107del] mutant maintains its ability to form small particles but has impaired ability to form larger particles. Our efflux experiments at both 6 and 24 h showed that a decreased amount of larger-sized nHDL was formed by the K107 mutant. After 24 h, apoA-I[K107del] demonstrated some ability to form some larger-sized nHDL particles predominantly less than 10.4 nm in size possibly driven by continued ABCA1 action but still significantly less compared to WT apoA-I. The detailed structural organization and stability of these particles and how apoA-I is organized on the particle is unknown but must be different to that of WT.

Additionally, of important note is that the apposition of helix 4 and helix 6 in the antiparallel double-belt forming the nHDL particle is thought to be the interaction site for LCAT. This apposition creates a cluster of charged residues that possibly form the LCAT-binding motif. Disruption of helix 4 by the K107 deletion together with changes in the organization of the charged cluster formed between helices 4 and 6 is likely to impact the LCAT interaction resulting in particles that are not only hindered in their formation but are dysfunctional in the important step of cholesterol esterification critical to RCT.

A suggested possible “hairpin-belt” model of apoA-I organization on larger discoidal particles assumes that the stabilizing salt-bridges in this model are identical to those in the “double-belt” model, except they are intra-helical versus interhelical for the “hairpin-belt” versus the double-belt, respectively (57). Thus, the disruption of the intra-helical and interhelical salt bridges in apoA-I resulting from deletion of Lys107 (15) would hinder the ability of apoA-I[K107del] to form the “hairpin-belt” structure as well on larger discoidal particles. Since K107del disrupts the intra-helical salt bridges in the vicinity of the deleted residue and the interhelical salt bridges between registered helices 4 and 6 (residues 99–120 and 143–164, respectively) of the two antiparallel apoA-I molecules in the “double-belt” (or in the same apoA-I molecule in the “hairpin” conformation), but likely does not disrupt salt bridges in the other helical regions of the apoA-I (15), apoA-I[K107del] can efficiently form smaller nHDL. It is likely that reduced binding of pro-apoA-I[K107del] to plasma HDL observed by Huang *et al.* (17) may relate to the impaired ability of the variant to bind to a population of larger HDL particles due the reasons described above.

In conclusion, the structural changes in apoA-I[K107del] may lead to 1) enhanced binding of the variant to and its better retention on plasma TG-rich lipoproteins and 2) impaired ability of apoA-I to form larger nHDL and stabilize larger spherical HDL. The former may result in an additional pool of plasma apoA-I on TG-rich lipoproteins that can contribute to normal plasma apoA-I levels, even with reduced HDL-bound apoA-I levels. The latter results in predominantly smaller plasma HDL particles with primarily reduced lipid:protein ratio and therefore, may contribute to the reduced plasma HDL-C levels along with normal apoA-I levels. In addition, the shift in the HDL size distribution resulting in a lower abundance of large HDL particles may lead to compromised antiatherogenic properties of HDL that are apparently influenced by particle size (reviewed in ref. (5)).

## Data Availability

All data is available upon request to the corresponding author.

## Supplemental Data

This article contains supplemental data.

## Conflict of Interest

The authors state no conflict of interest.

## Supplemental data

Figure S1

Figure S2A-B

Figure S2C

## References

[1] Phillips M.C. (2013). New insights into the determination of HDL structure by apolipoproteins: thematic Review Series: high density lipoprotein structure, function, and metabolism. J. Lipid Res..

[2] Dominiczak M.H., Caslake M.J. (2011). Apolipoproteins: metabolic role and clinical biochemistry applications. Ann. Clin. Biochem..

[3] Sharrett A.R., Ballantyne C.M., Coady S.A., Heiss G., Sorlie P.D., Catellier D. (2001). Coronary heart disease prediction from lipoprotein cholesterol levels, triglycerides, lipoprotein(a), apolipoproteins A-I and B, and HDL density subfractions: the Atherosclerosis Risk in Communities (ARIC) Study. Circulation.

[4] Rader D.J., Tall A.R. (2012). The not-so-simple HDL story: is it time to revise the HDL cholesterol hypothesis?. Nat. Med..

[5] Rye K.A., Barter P.J. (2014). Cardioprotective functions of HDLs. J. Lipid Res..

[6] Pownall H.J., Rosales C., Gillard B.K., Gotto A.M., M A. (2021). High-density lipoproteins, reverse cholesterol transport and atherogenesis. Nat. Rev. Cardiol..

[7] Rosenson R.S., Brewer H.B., Davidson W.S., Fayad Z.A., Fuster V., Goldstein J. (2012). Cholesterol efflux and atheroprotection: advancing the concept of reverse cholesterol transport. Circulation.

[8] Sorci-Thomas M.G., Thomas M.J. (2002). The effects of altered apolipoprotein A-I structure on plasma HDL concentration. Trends Cardiovasc. Med..

[9] Asztalos B.F., Demissie S., Cupples L.A., Collins D., Cox C.E., Horvath K.V. (2006). LpA-I, LpA-I: A-II HDL and CHD-risk: the Framingham Offspring Study and the Veterans Affairs HDL Intervention Trial. Atherosclerosis.

[10] Haase C.L., Frikke-Schmidt R., Nordestgaard B.G., Tybjærg-Hansen A. (2012). Population-based resequencing of APOA1 in 10,330 individuals: spectrum of genetic variation, phenotype, and comparison with extreme phenotype approach. PLoS Genet..

[11] Tilly-Kiesi M., Lichtenstein A.H., Ordovas J.M., Dolnikowski G., Malmström R., Taskinen M.-R. (1997). Subjects with ApoA-I(Lys107→0) exhibit enhanced fractional catabolic rate of ApoA-I in Lp(AI) and ApoA-II in Lp(AI With AII). Atheroscler. Thromb. Vasc. Biol..

[12] Nofer J.R., von Eckardstein A., Wiebusch H., Weng W., Funke H., Schulte H. (1995). Screening for naturally occurring apolipoprotein A-I variants: apo A-I(delta K107) is associated with low HDL-cholesterol levels in men but not in women. Hum. Genet..

[13] Amarzguioui M., Mucchiano G., Häggqvist B., Westermark P., Kavlie A., Sletten K. (1998). Extensive intimal apolipoprotein A1-derived amyloid deposits in a patient with an apolipoprotein A1 mutation. Biochem. Biophys. Res. Commun..

[14] Rowczenio D., Dogan A., Theis J.D., Vrana J.A., Lachmann H.J., Wechalekar A.D. (2011). Amyloidogenicity and clinical phenotype associated with five novel mutations in apolipoprotein A-I. Am. J. Pathol..

[15] Gorshkova I.N., Mei X., Atkinson D. (2014). Binding of human apoA-I[K107del] variant to TG-rich particles: implications for mechanisms underlying hypertriglyceridemia. J. Lipid Res..

[16] Ramella N.A., Schinella G.R., Ferreira S.T., Prieto E.D., Vela M.E., Ríos J.L. (2012). Human apolipoprotein A-I natural variants: molecular mechanisms underlying amyloidogenic propensity. PLoS One..

[17] Huang W., Matsunaga A., Li W., Han H., Hoang A., Kugi M. (2001). Recombinant proapoA-I(Lys107del) shows impaired lipid binding associated with reduced binding to plasma high density lipoprotein. Atherosclerosis.

[18] Tilly-Kiesi M., Zhang Q., Ehnholm S., Kahri J., Lahdenperä S., Ehnholm C. (1995). ApoA-I_Helsinki_ (Lys107-->0) associated with reduced HDL cholesterol and LpA-I: A-II deficiency. Arterioscler. Thromb. Vasc. Biol..

[19] Tilly-Kiesi M., Packard C.J., Kahrij J., Ehnholm C., Shepherd J., Taskinen M.R. (1997). In vivo metabolism of apoA-I and apo A-II in subjects with apo A-I(Lys107-->0) associated with reduced HDL cholesterol and Lp(AI w AII) deficiency. Atherosclerosis.

[20] Curtiss L.K., Valenta D.T., Hime N.J., Rye K.A. (2006). What Is So Special About Apolipoprotein AI in Reverse Cholesterol Transport?. Arterioscler. Thromb. Vasc. Biol..

[21] Wang L., Atkinson D., Small D.M. (2005). The interfacial properties of ApoA-I and an amphipathic alpha-helix consensus peptide of exchangeable apolipoproteins at the triolein/water interface. J. Biol. Chem..

[22] Meyers N.L., Wang L., Gursky O., Small D.M. (2013). Changes in helical content or net charge of apolipoprotein C-I alter its affinity for lipid/water interfaces. J. Lipid Res..

[23] Wang L., Mei X., Atkinson D., Small D.M. (2014). Surface behavior of apolipoprotein A-I and its deletion mutants at model lipoprotein interfaces. J. Lipid Res..

[24] Small D.M., Wang L., Mitsche M.A. (2009). The adsorption of biological peptides and proteins at the oil/water interface. A potentially important but largely unexplored field. J. Lipid Res..

[25] Meyers N.L., Wang L., Small D.M. (2012). Apolipoprotein C-I binds more strongly to phospholipid/triolein/water than triolein/water interfaces: a possible model for inhibiting cholesterol ester transfer protein activity and triacylglycerol-rich lipoprotein uptake. Biochemistry.

[26] Mitsche M.A., Wang L., Small D.M. (2010). Adsorption of egg phosphatidylcholine to an air/water and triolein/water bubble interface: use of the 2-dimensional phase rule to estimate the surface composition of a phospholipid/triolein/water surface as a function of surface pressure. J. Phys. Chem. B..

[27] von Eckardstein A., Castro G., Wybranska I., Theret N., Duchateau P., Duverger N. (1993). Interaction of reconstituted high density lipoprotein discs containing human apolipoprotein A-I (apoA-I) variants with murine adipocytes and macrophages. J.Biol. Chem..

[28] Gonzalez M.C., Toledo J.D., Tricerri M.A., Garda H.A. (2008). The central type Y amphipathic a-helices of apolipoprotein AI are involved in the mobilization of intracellular cholesterol depots. Arch. Biochem. Biophys..

[29] Sankaranarayanan S., Kellner-Weibel G., de la Llera-Moya M., Phillips M.C., Asztalos B.F., Bittman R. (2011). A sensitive assay for ABCA1-mediated cholesterol efflux using BODIPY-cholesterol. J. Lipid Res..

[30] Mei X., Liu M., Herscovitz H., Atkinson D. (2016). Probing the C-terminal domain of lipid-free apoA-I demonstrates the vital role of the H10B sequence repeat in HDL formation. J. Lipid Res..

[31] Liu M., Mei M., Herscovitz H., Atkinson D. (2019). N-terminal mutation of apoA-I and interaction with ABCA1 reveal mechanisms of nascent HDL biogenesis. J. Lipid Res..

[32] Duong P.T., Collins H.L., Nickel M., Lund-Katz S., Rothblat G.H., Phillips M.C. (2006). Characterization of nascent HDL particles and microparticles formed by ABCA1-mediated efflux of cellular lipids to apoA-I. J. Lipid Res..

[33] Ludovico I.D., Gisonno R.A., Gonzalez M.C., Garda H.A., Ramella N.A., Tricerri M.A. (2021). Understanding the role of apolipoproteinA-I in atherosclerosis. Post-translational modifications synergize dysfunction?. Biochim. Biophys. Acta Gen. Subj..

[34] Gorshkova I.N., Liu T., Kan H.Y., Chroni A., Zannis V.I., Atkinson D. (2006). Structure and stability of apolipoprotein A-I in solution and in discoidal high-density lipoprotein probed by double charge ablation and deletion mutation. Biochemistry.

[35] Mendivil C.O., Furtado J., Morton A.M., Wang L., Sacks F.M. (2016). Novel pathways of apolipoprotein A-I metabolism in HDL of different sizes in humans. Arterioscler. Thromb. Vasc. Biol..

[36] Bedi S., Morris J., Shah A., Hart R.C., Jerome W.G., Aller S.G. (2022). Conformational flexibility of apolipoprotein A-I amino- and carboxy-termini is necessary for lipid binding but not cholesterol efflux. J. Lipid Res..

[37] Rall S.C., Weisgraber K.H., Mahley R.W., Ogawa Y., Fielding C.J., Utermann G. (1984). Abnormal lecithin:cholesterol acyltransferase activation by a human apolipoprotein A-I variant in which a single lysine residue is deleted. J. Biol. Chem..

[38] Jonas A., von Eckardstein A., Churgay L., Mantulin W.W., Assmann G. (1993). Structural and functional properties of natural and chemical variants of apolipoprotein A-I. Biochim. Biophys. Acta.

[39] Ludovico I.D., Bedi S., Melchior J.T., Gonzalez M., Garda H., Davidson S. (2020). Deletion of Lys 107 Modify the Ability of ApoA-I to Self-associate in Solution. Arterioscler. Thromb. Vasc. Biol..

[40] Haase C.L., Tybjærg-Hansen A., Grande P., Frikke-Schmidt R. (2010). Genetically elevated apolipoprotein A-I, high-density lipoprotein cholesterol levels, and risk of ischemic heart disease. J. Clin. Endocrinol. Metab..

[41] Weisgraber K.H., Rall S.C., Bersot T.P., Mahley R.W., Franceschini G.,, Sirtori C.R. (1983). Apolipoprotein A-I milano - detection of normal A-I in affected subjects and evidence for a cysteine for arginine substitution in the variant A-I. J. Biol. Chem..

[42] Anthanont P., Polisecki E., Asztalos B.F., Diffenderfer M.R., Barrett P.H., Millar J.S. (2014). A novel ApoA-I truncation (ApoA-I _Mytilene_) associated with decreased ApoA-I production. Atherosclerosis.

[43] Mei X., Atkinson D. (2011). Crystal structure of C-terminal truncated apolipoprotein A-I reveals the assembly of high density lipoprotein (HDL) by dimerization. J. Biol. Chem..

[44] Melchior J.T., Walker R.G., Cooke A.L., Morris J., Castleberry M., Thompson T.B. (2017). A consensus model of human apolipoprotein A-I in its monomeric and lipid-free state. Nat. Struct. Mol. Biol..

[45] Chroni A., Kan H.Y., Kypreos K.E., Gorshkova I.N., Shkodrani A., Zannis V.I. (2004). Substitutions of Glu110 and Glu111 in the middle helix 4 of human ApoA-I by Alanine affect the structure and in vitro functions of apoA-I and induce severe hypertriglyceridemia in ApoA-I-deficient mice. Biochemistry.

[46] Kateifides A.K., Gorshkova I.N., Duka A., Chroni A., Kardassis D., Zannis V.I. (2011). Alteration of negatively charged residues in the 89 and 96 domain of apoA-I affects lipid homeostasis and maturation of HDL.. J. Lipid Res..

[47] Gorshkova I.N., Atkinson D. (2011). Enhanced Binding of Apolipoproteins A-I Variants Associated with Hypertriglyceridemia to Triglyceride-Rich Particles. Biochemistry.

[48] Chroni A., Liu T., Gorshkova I., Kan H.Y., Uehara Y., von Eskardstein A. (2003). The central helices of apoA-I can promote ATP-binding cassette transporter A1 (ABCA1)-mediated lipid efflux. Amino acid residues 220-231 of the wild-type apoA-I are required for lipid-efflux in vitro and high density lipoprotein formation in vivo. J. Biol.Chem..

[49] Arakawa R., Yokoyama S. (2002). Helical apolipoproteins stabilize ATP-binding cassette transporter A1 by protecting it from thiol protease-mediated degradation. J. Biol. Chem..

[50] Vedhachalam C., Chetty P.S., Nickel M., Dhanasekaran P., Lund-Katz S., Rothblat G.H. (2010). Influence of apolipoprotein (Apo) A-I structure on nascent high density lipoprotein (HDL) particle size distribution. J. Biol. Chem..

[51] Liu L., Bortnick A.E., Nickel M., Dhanasekaran P., Subbaiah P.V., Lund-Katz S. (2003). Effects of apolipoprotein A-I on ATP-binding cassette transporter A1-mediated efflux of macrophage phospholipid and cholesterol: formation of nascent high density lipoprotein particles. J. Biol. Chem..

[52] Nilsson O., Lindvall M., Obici L., Ekström S., Lagerstedt J.O., Del Giudice R.J. (2021). Structure dynamics of ApoA-I amyloidogenic variants in small HDL increase their ability to mediate cholesterol efflux. J. Lipid Res..

[53] Silva R.A., Huang R., Morris J., Fang J., Gracheva E.O., Ren G. (2008). Structure of apolipoprotein A-I in spherical high density lipoproteins of different sizes. Proc. Natl. Acad. Sci. U. S. A..

[54] Huang R., Silva R.A., Jerome W.G., Kontush A., Chapman M.J., Curtiss L.K. (2011). Apolipoprotein A-I structural organization in high-density lipoproteins isolated from human plasma. Nat. Struct. Mol. Biol..

[55] Segrest J.P., Jones M.K., Klon A.E., Sheldahl C.J., Hellinger M., De Loof H. (1999). A detailed molecular belt model for apolipoprotein A-I in discoidal high density lipoprotein. J. Biol. Chem..

[56] Li L., Chen J., Mishra V.K., Kurtz J.A., Cao D., Klon A.E. (2004). Double belt structure of discoidal high density lipoproteins: molecular basis for size heterogeneity. J. Mol. Biol..

[57] Davidson W.S., Silva R.A. (2005). Apolipoprotein structural organization in high density lipoproteins: belts, bundles, hinges and hairpins. Curr. Opin. Lipidol..

[58] Chetty P.S., Mayne L., Kan Z.-Y., Lund-Katz S., Englander S.W., Phillips M.C. (2012). Apolipoprotein A-I helical structure and stability in discoidal high-density lipoprotein (HDL) particles by hydrogen exchange and mass spectrometry. Proc. Natl. Acad. Sci. U. S. A..

[59] Sparks D.L., Lund-Katz S., Phillips M.C. (1992). The charge and structural stability of apolipoprotein A-I in discoidal and spherical recombinant high density lipoprotein particles. J. Biol. Chem..

