# Supplementary figures and images for: Human apoA-I[Lys107del] mutation affects lipid surface behavior of apoA-I and its ability to form large nascent HDL
Source: J Lipid Res. 2022 Dec 13;64(2):100319. doi: 10.1016/j.jlr.2022.100319 (PMC9926306; doi:10.1016/j.jlr.2022.100319)

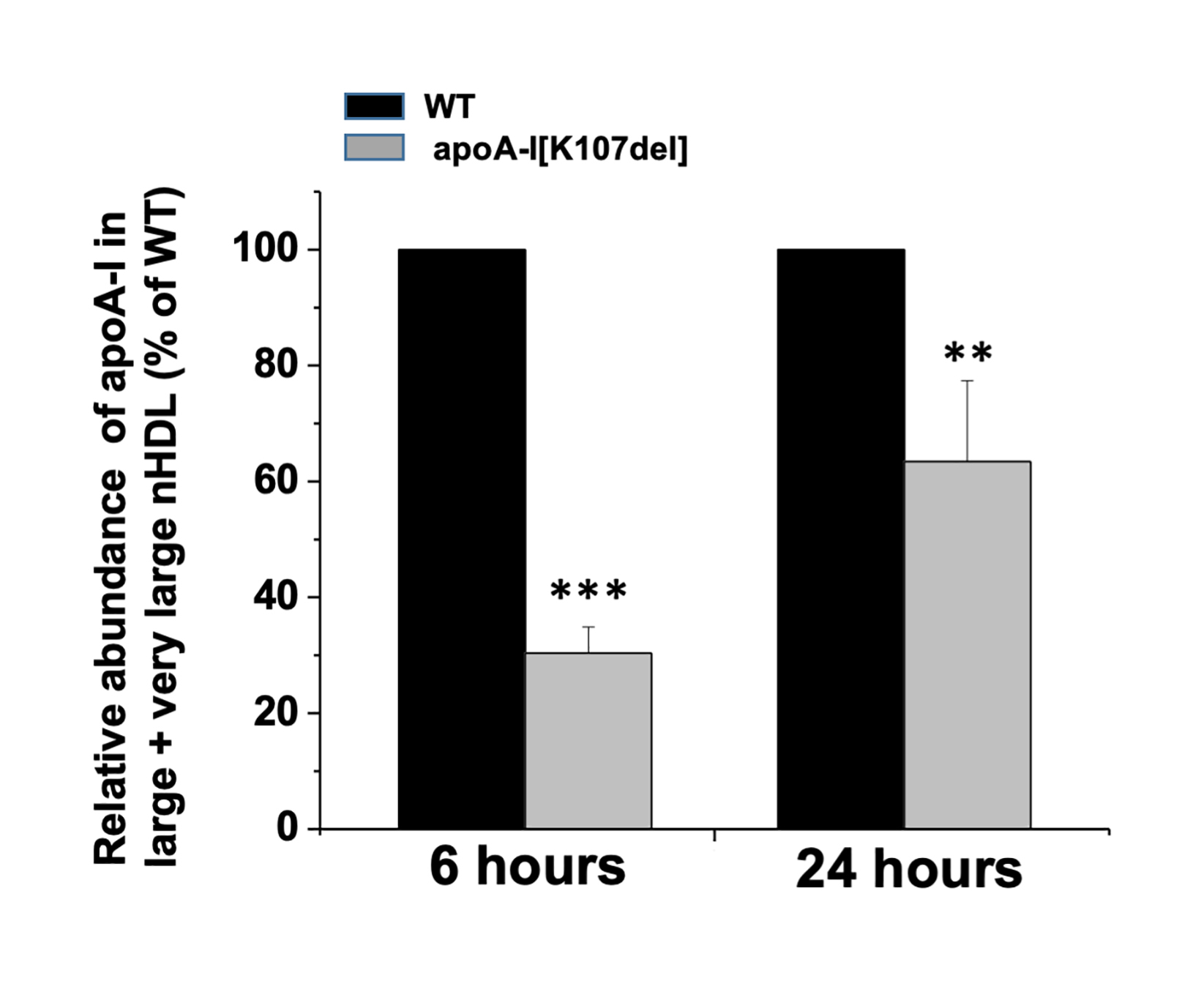

Supplement: Figure S1 [file figs1.jpg]

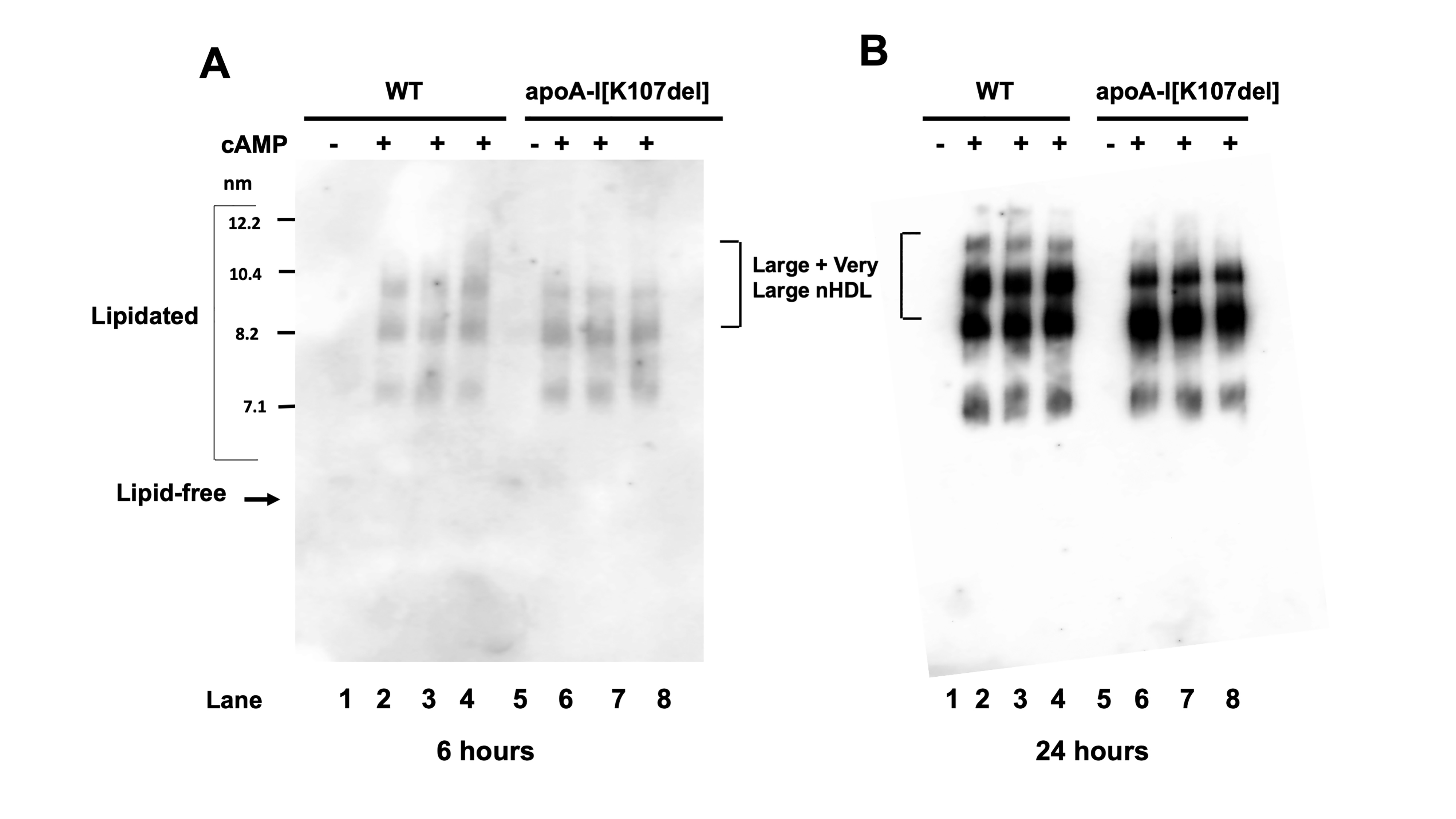

Supplement: Figure S2A-B [file figs2.jpg]

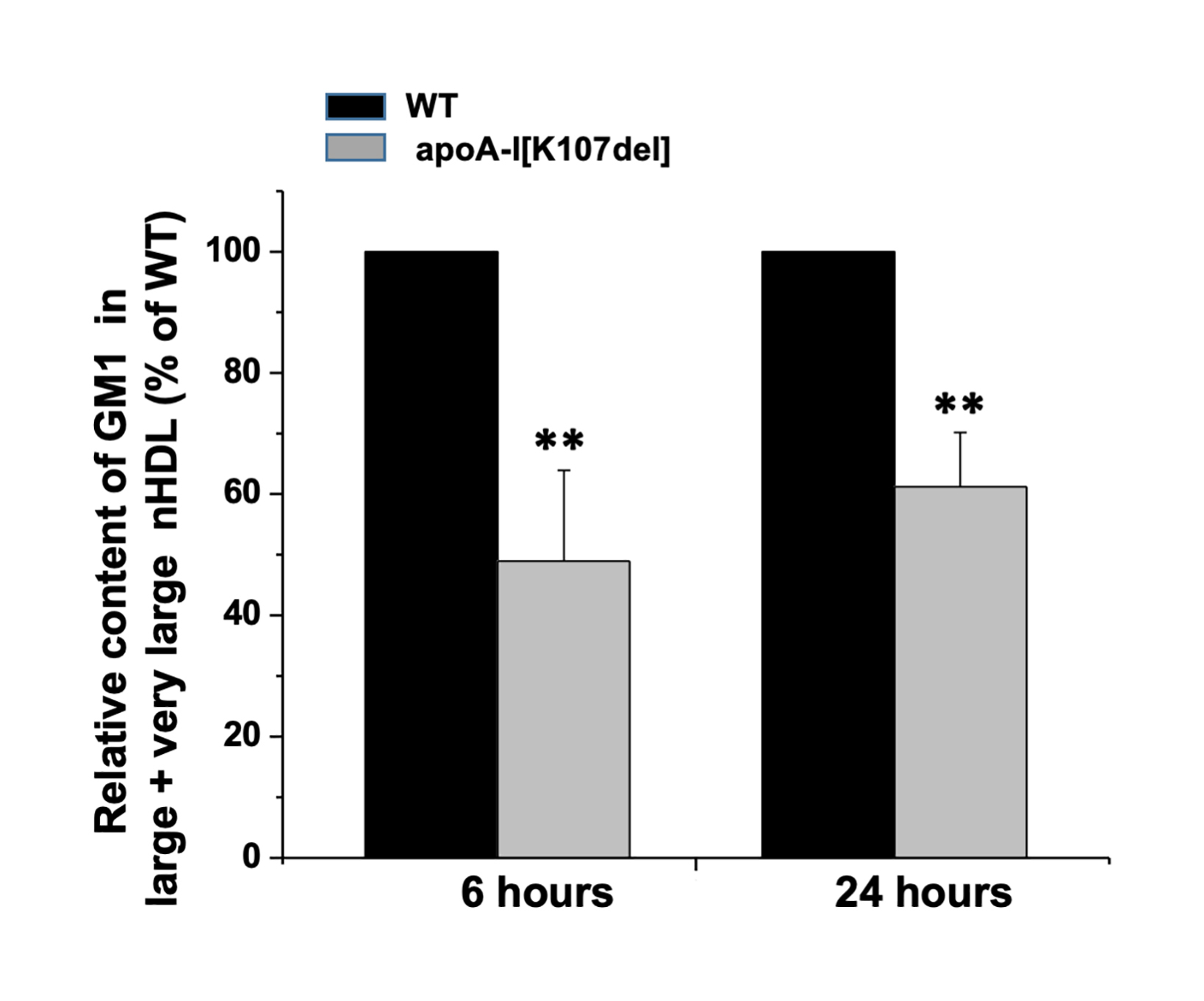

Supplement: Figure S2C [file figs3.jpg]
