# Supplementary material for: Human apoA-I[Lys107del] mutation affects lipid surface behavior of apoA-I and its ability to form large nascent HDL
Source: J Lipid Res. 2022 Dec 13;64(2):100319. doi: 10.1016/j.jlr.2022.100319 (PMC9926306; doi:10.1016/j.jlr.2022.100319)
Supplement: Legends for Supplemental Figures [file mmc1.docx]

**Supplemental Figure S1**. **Relative abundance of large plus very large nHDL ( > 8.5 nm-diameter) formed after 6- or 24-h incubation**. Bands corresponding to large and very large nHDL depicted in **Figures 4C and 4D** were quantified, and their combined values were expressed as % of total nHDL. Values for apoA-I[K107del] (grey bars) were expressed as percentage of WT (set to 100%, black bars). Bars represent means ± SD (n = 5). ** P < 0.01; *** P < 0.001.

**Supplemental Figure S2.**  Analysis of GM1 content in nHDL. A: and B: Ligand blots showing GM1 in nHDL formed after 6- and 24-h incubation, respectively. The membranes shown in **Fig. 4C, D** were stripped and incubated with cholera toxin subunit B to detect GM1. C: Relative abundance of GM1 in combined large plus very large nHDL (>8.5 nm-diameter). Bands marked as large and very large nHDL-apoA-I depicted in **panels A and B** were quantified, and expressed as a percentage of total nHDL. Values for apoA-I[K107del] (grey bars) were expressed as percentage of WT (set to 100%, black bars). Bars represent means ± SD (n = 4). ** P < 0.01.
